# MAP4Ks drive cell death in response to *Salmonella* SpvB-induced actin depolymerization

**DOI:** 10.1128/mbio.00655-26

**Published:** 2026-05-20

**Authors:** Mitchell A. Pallett, Romina Tocci, Andrea Majstorovic, Elena Everatt, Ioanna Panagi, Ines Diaz-del-Olmo, Camilla Godlee, Teresa L. M. Thurston, David W. Holden

**Affiliations:** 1 Centre for Bacterial Resistance Biology, Department of Infectious Disease, Faculty of Medicine, Imperial College London 4615, London, United Kingdom; 2 Sir William Dunn School of Pathology, University of Oxford, Oxford, United Kingdom; 3 Department of Biochemistry, Cambridge University, Cambridge, United Kingdom; University of Pennsylvania Perelman School of Medicine, Philadelphia, Pennsylvania, USA

**Keywords:** cell death, actin, pathogenesis, host–pathogen interactions

## Abstract

Many pathogens target the host actin cytoskeleton through the delivery of actin depolymerizing toxins, including mono-ADP-ribosyltransferases (mART), ultimately triggering host cell death. Despite the importance of mARTs in pathogen virulence, it remains unclear whether actin ribosylation is required for mART-dependent cell death, and how actin depolymerization leads to cell death. Using the non-typhoidal *Salmonella enterica* Typhimurium-encoded mART, SpvB, we report that cell death is induced exclusively through ribosylation of actin. We found cell death to be morphologically and mechanistically distinct from apoptosis as well as any previously reported mode of cell death. Instead, our data identify the Hippo signaling MAP4Ks as the essential host cell sensors of actin depolymerization signaling through JNK to facilitate vacuolization and host cell death. Cell death following treatment of cells with the actin depolymerizing agent latrunculin A followed the same pathway, identifying a conserved mechanism of cell death. Therefore, we identify MAP4K family members as key regulators of an atypical caspase-independent cell death induced by actin depolymerization, building on our understanding of host-cell death signaling and mechanisms of bacterial virulence.

**IMPORTANCE:**

Host cell death plays a critical role as an intrinsic defense mechanism against infection and disease. However, many pathogens subvert cell death signaling to enhance their replication and survival. Here, we show that the mono-ADP ribosyl transferase family of toxins encoded by pathogens of global importance, including *Salmonella* spp., *Neisseria* spp. and *C. difficile* induces actin depolymerization leading to MAP4K activation and JNK-dependent cell death. Through mechanistically characterizing this atypical cell death pathway, our study identifies and positions key components of a previously undescribed cell death pathway and broadens our understanding of bacterial pathogenesis and virulence.

## INTRODUCTION

Actin, the most abundant protein in most mammalian cells, has a crucial role in essential processes, including cellular adhesion, cell–cell junctions, migration, cell division, cell shape, polarization, and ultimately cell survival (1–4). Actin regulates these diverse pathways through transitioning between its monomeric (G-actin) and polymerized (F-actin) state. This transition is regulated by ATP hydrolysis of F-actin and further regulated by actin-binding proteins (ABPs) that influence the steady state (5, 6). Given its key role in these processes, it is unsurprising that actin polymerization is subverted by bacterial, viral, and parasitic pathogens. Targeting the actin cytoskeleton facilitates pathogen entry, spread, escape from vacuoles, and establishment of intracellular niches for pathogen survival (7, 8). The focus of the majority of these studies has been on the sensing of pathways facilitating actin polymerization, but many pathogens deliver cytotoxins that specifically drive actin depolymerization and destruction of the cell actin cytoskeleton, leading to host cell death (9). Despite the importance of these toxins in pathogen virulence, it remains unclear how the host senses changes in polymerized actin and what the intracellular signals are leading to cell death.

Actin depolymerizing agents and toxins are found across the Kingdoms, ranging from bacteria and fungi to sponges. The most well-known actin depolymerizing agents are the naturally cell-permeable alkaloids latrunculin A (LatA) and cytochalasin D. Both are routinely used at low concentrations in cell assays to study pathways regulated by actin polymerization. Mechanistically, LatA inhibits polymerization by sequestration of G-actin and inhibition of nucleotide exchange (10), whereas cytochalasin D binds the barbed (+) end of actin filaments and inhibits ATP-hydrolysis in G-actin dimers to prevent polymerization and elongation (6). Of the bacterial toxins known to target actin for depolymerization, the mono-ADP-ribosyltransferase (mART) family is the largest and is encoded by the bacterial pathogens *Clostridium botulinum* (C2 toxin) (11), *Clostridium perfringens* (Iota toxin/ CPILE-a/ BECa) (12, 13), *Clostridioides difficile* (CDTa) (14), *Aeromonas hydrophila* (VrgG1/VahC) (15), *Salmonella enterica* Serovar Typhimurium (STm)/Enteritidis/Paratyphi B/C (SpvB) (16), *Bacillus cereus* (VIP2) (17), *Neisseria meningitidis* (NarE) (18), and *Photorhabdus luminescens* (TcC; Photox) (19, 20). Commonly, the mART activity is expressed as a bipartite/tripartite complex, which enables toxin delivery. For example, C2 is encoded by C2-I and C2-II. C2-I encodes the mART enzymatic activity, whereas C2-II is required for cell surface binding, uptake, pore formation, and cytoplasmic delivery of C2-I (21). In contrast, as an intracellular pathogen, vacuolar STm translocates SpvB into the host cell cytoplasm through a needle-like syringe known as the SPI-2 injectisome or Type III Secretion System (T3SS) (22). The only known target of the mART activity of these toxins is monomeric G-actin. G-actin is ADP-ribosylated at R177 specifically (23, 24), and when incorporated into actin filaments, the modified ADP-actin prevents further polymerization, triggering depolymerization and inducing collapse of the host cell cytoskeleton (25). Delivery of recombinant or ectopically expressed mARTs, including C2, SpvB, VahC, NarE, and Iota, is reported to induce caspase activation and DNA fragmentation: hallmarks of caspase-dependent apoptosis (9, 15, 18, 26, 27). Despite these observations, a direct link between caspase activation and actin depolymerization-induced cell death has not been established. In fact, recent findings that inhibition of caspase activity does not rescue DNA fragmentation in response to SpvB and C2 suggest that an alternative or parallel caspase-independent mechanism drives cell death (26). Of the mARTs, SpvB is the most well characterized and has only one reported enzymatic function: ADP-ribosylation of G-actin (28, 29). This makes it a model mART to study mechanisms of actin depolymerization-induced cell death.

SpvB, which is associated with bacteremia and disease severity in humans and contributes to virulence in the mouse model of systemic infection, is encoded by the *Salmonella* virulence plasmid (*spv*) locus, which contains five genes: *spvA-D* and a regulator *spvR* (28, 30). SpvB has distinct N-terminal and C-terminal domains separated by a proline repeat bridge (16). The N-terminal region shares similarity with *P. luminescens* TcaC (required for toxin delivery) (19) but lacks the full domain required for pore formation and uptake, and it is dispensable for the mART activity of SpvB, which is encoded in the C-terminal domain (16, 31). Many studies have identified numerous cellular processes subverted by SpvB, including the induction of cell cycle arrest (27), inhibition of autophagy (32), inhibition of NF-κB activity (33), disassembly of P-body formation (34), disruption of iron homeostasis (35), and induction of cell death (30). Whether the dysregulation of these pathways is directly or indirectly due to SpvB-dependent depolymerization of actin requires further investigation. However, complementation of the virulence defect of the STm *spvB* mutant strain *in vivo* is dependent upon SpvB mART-activity, suggesting G-actin ADP-ribosylation is key to the function of SpvB and the virulence of STm (28). Consistent with the other mARTs, cell death caused by ectopic expression of SpvB is characterized by cell rounding, cell detachment, and DNA damage (26, 27). STm is reported to induce cell death in a SPI-2 T3SS-dependent manner (as recently reviewed (36)); however, it is unclear whether this is SpvB-dependent or not. Of note, SpvB-treated cells undergo cell death that is morphologically distinct from caspase-dependent cell death, with cell rounding, swelling, and vacuolization in comparison to cell shrinkage and membrane blebbing (27). This, along with previous reports of caspase inhibition failing to rescue SpvB-induced DNA damage, suggests that SpvB-induced cell death is distinct from the proposed mechanism of caspase-3-dependent apoptosis (26).

Cell death signaling during STm infection is complex, with activation of multiple pathways and inhibition of one typically leading to activation of an alternative cell death rather than complete rescue (37, 38). Many studies have focused on the regulation of cell death in macrophages, given the importance of this cellular niche for STm replication. Here, cell death is primarily driven by pyroptosis through caspase-11/cathepsin-dependent non-canonical inflammasome activation in response to cytoplasmic LPS detection and NLRP3/NLRC4 canonical inflammasome activation (39–42). Although pyroptosis plays a major role in cell death during STm infection, multiple independent studies have revealed activation of necroptosis in the absence or inhibition of caspase activation (43–45). This commitment to cell death by multiple pathways has been termed PANoptosis: death by any means (37). Inactivation of the SPI-2 T3SS severely hampers STm-induced macrophage cell death; however, although SpvB and another effector, SseL, have been implicated in macrophage cell death, the mechanisms remain unknown (42, 46–48).

Cell death in non-hematopoietic cells follows different pathways in response to STm infection. In enterocytes, NLRC4/caspase-1-dependent inflammasomes are implicated in early responses, whereas caspase-11 plays a compensatory role later in infection following IFN-γ priming (49, 50). Contrary to macrophages, activation of pyroptosis in enterocytes drives cell extrusion prior to death (50, 51). SpvB has also been implicated in enterocyte cell death *in vivo* (52). Infection with the Δ*spvB* strain of STm in mouse models reduced TUNEL-positive enterocyte staining and rescued intestinal barrier integrity, supporting a role for SpvB in enterocyte cell death *in vivo* (52, 53). Although the authors concluded that this cell death is RIPK3/MLKL-dependent (52), a previous study reported that enterocyte MLKL was protective against infection through limiting bacterial colonization (54), while another demonstrated that intestinal epithelial cell death during STm infection of mice was unaffected by knockout of *Ripk3* or *Mlkl* (55). Therefore, a role for SpvB in enterocyte RIPK3-dependent necroptosis in infection is unclear and requires further investigation.

In this work, we set out to characterize the host cell pathways and mechanisms regulating cell death following actin depolymerization using SpvB as a model mART.

## MATERIALS AND METHODS

### Cells, plasmids, bacterial strains, and reagents

All reagents were purchased from Sigma-Aldrich unless otherwise stated. HEK293ET (ATCC CRL-3216, human embryonic kidney cells), HeLa (93021013), immortalized bone marrow–derived macrophages (iBMDMs) (56), J774.1A (91051511), and MelJuso cells were maintained in Dulbecco’s modified Eagle’s medium (DMEM; D5796) high glucose and supplemented with 10% fetal bovine serum (FBS; Invitrogen) and non-essential amino acids (NEAA; TMS-001) and incubated at 37°C in a 5% CO_2_ atmosphere. iBMDMs were further supplemented with 20% L929 conditioned medium as described previously (56). HEK293A WT and HEK293A KO (*MAP4K1/2/3/4/6/7^−/−^* and *MST1/2^−/−^*) were kind gifts from Prof. Dario Alessi, University of Dundee, and have been described previously (57). All plasmids used and those constructed during this study are listed in Table S1. Plasmids were generated using conventional restriction enzyme (RE) digest and ligation or one-step sequence- and ligation-independent cloning (SLIC) (58) using the primers and RE sites listed in Tables S1 and S2. The SpvB catalytically dead mutant (SpvB E536A/E538A; SpvBm) (26) was generated by three-stage overlapping extension PCR using primer sets 1/2 and 3/4 (Table S2) to generate templates for PCR with primer set 1/6 and 4/5, respectively, before combining templates for PCR with primer set 1/4 for the final template for cloning into plasmids indicated in Table S1. The Actin R177G mutant plasmid was constructed similarly, using two-stage overlapping PCR using primer set 9/10 and 11/12 (Table S2) before a final PCR with primer set 9/12 for cloning into pCDNA4/TO. Strains used in this study are listed in Table S3. MAP4K4/6/7 inhibitor PF-6260933 (R&D Systems; 5752) was diluted in water and used at 40 μM. The pan-caspase inhibitor zVAD-FMK (R&D Systems; FMK001), calpain and cathepsin B/L inhibitor ALLM (Santa Cruz; sc-201268), and JNK inhibitor SP600125 (Merck Chemicals; 420,128) were re-suspended in DMSO and used at 50 μM, 30 μM, and 25 μM, respectively. Pepstatin A (Bio-Techne; 1190) was resuspended in methanol, gently warmed at 60°C, and used at 15 μM. Latrunculin A (R&D Systems; 3973) was resuspended in DMSO and used at 9.6 μM.

### Preparation of lentiviral particles and transduction of target cells

The pLTREK-puro plasmids listed in Table S1 were co-transfected with the packaging (psPAX) and envelope (pMD2.6) plasmids for VsVg pseudotyped lentivirus production in HEK293ETs using Lipofectamine 2000 transfection reagent (Invitrogen: 11668019). After 48 h, the medium was centrifuged at 300 *× g*, filtered, aliquoted, and frozen at −80°C. Target cells, including HCT116, HEK293, MelJuso, HeLa, J774A.1, and iBMDM, were transduced with the specific packaged replication-defective lentiviral particles, with 8 μg/mL Polybrene (TR-1003-G) and 1 μg/mL doxycycline (D9891) to induce protein expression. Transduction and expression were confirmed by immunoblotting of cell lysates.

### Generation of HEK293A Flag-actin stable cell lines

pCDNA4/TO-EV, pCDNA4/TO-Flag-actin, and pCDNA/TO-Flag-actin R177G were transfected into HEK293A using Lipofectamine 2000. Twenty-four hours post-transfection, cells were selected in 100 μg/mL zeocin (ThermoFisher; R25001). Cells were maintained in zeocin for 2 weeks before culturing in the absence of Zeocin. Expression of Flag-actin and Flag-actin-R177G was confirmed by immunoblotting.

### Generation of stable shRNA-expressing cell lines

Target shRNAs were selected using the online tool DSIR (Designer of small interfering siRNA) (59). Unique miR30E inserts were generated using primer set 7/8 (Table S2) along with *MAP4K4*-, *AIF-*, *ENDOG-,* or *CASP3*-specific primers in Table S1, as described previously, and subcloned directly into pMXs-IP-YFP using SLIC and RE sites EcoRI and XhoI (60). Specific 22-mer sequences are shown in Table S1. The pMXs-IP-YFP-shRNA plasmids listed in Table S1 were co-transfected with the packaging (pCMV-PACK) and envelope (pMD2.6) plasmids for VsVg pseudotyped retrovirus production in HEK293ETs using Lipofectamine 2000 transfection reagent (Invitrogen). After 48 h, the medium was centrifuged at 300 *× g*, filtered, aliquoted, and frozen at −80°C. Target cells were transduced with the specific packaged replication-defective retroviral particles and selected in 1 μg/mL Puromycin (P8833) before sorting for the high-YFP expressing population by FACS using the BD FACS Aria III Cell sorter (BD Biosciences). Knockdown efficiency was confirmed by immunoblotting.

### Generation of JNK knockout cell lines

Guide DNAs for JNK1 (*MAPK8*), JNK2 (*MAPK9*), and JNK3 (*MAPK10*; Table S1.) were subcloned into pX330, and plasmids carrying two unique guides per gene were transfected into HEK293A cells, using Lipofectamine 2000 following the manufacturer’s instructions. Selection was carried out as described previously (61). Clones were screened for knockout of *MAPK8/9/10* (JNK1/2/3) and *MAPK10* (JNK3) by immunoblotting with a JNK3-specific antibody (Santa Cruz; sc-130075) and a pan-JNK antibody (Santa Cruz; sc-7345) targeting all three isoforms.

### Transfection

HeLa cells were seeded in 96-well plates (wp), 24 wp, or 6 wp at 1 × 10^4^, 5 × 10^4^, or 2.5 × 10^5^, respectively. Eighteen hours later, cells were transfected with 150 ng of pCDNA4/TO-EV, 150 ng of ptCMV-GFP-SpvB, or 75 ng of pEGFP-N1 with 75 ng of pCDNA4/TO-EV per 96-well plate using Lipofectamine 2000 following the manufacturer’s protocol. Six hours post-transfection, the media was replaced with Opti-MEM (Invitrogen; 11058-021) supplemented with 10% FCS. HEK293A WT or KO cells were seeded in 96 wp, 24 wp, or 6 wp at 1.5 × 10^4^, 7.5 × 10^4^, or 3.75 × 10^5^, respectively. Eighteen hours later, cells were transfected with 150 ng of pCDNA4/TO-EV, 150 ng of ptCMV-GFP-SpvB, or 75 ng of pEGFP-N1 with 75 ng of pCDNA4/TO-EV per 96-well plate using Lipofectamine 2000. Where dose responses were analyzed, plasmids were added using the same ratios as above but adding 50%, 33%, or 16.7% of the expression vector. For all conditions, the same total amount of plasmid was added by supplementing with pCDNA4/TO-EV. Four hours post-transfection, the media was replaced with Opti-MEM supplemented with 10% FCS. Cells were analyzed 24 h post-transfection, where transfection efficiency, as seen by IFA, was near 100%. Cell death and detachment assays were set up with four technical replicates per condition.

### Cell detachment assays

Following transfection or treatment, the media was collected, and the cells were pelleted at 400 *× g* for 5 min. The supernatant was removed, and the pellet was gently resuspended in PBS. The absolute cell number was calculated by counting the cell number on a hemocytometer. Assays were conducted with at least three technical replicates.

### Light micrographs

Twenty-four hours post-transfection or 18 h post-LatA treatment, cell monolayers were imaged using an Olympus CKX41 light microscope with an 8-megapixel iSight camera (Apple). Images were taken at 5× zoom, sectioned into quadrants, and cell morphology was recorded and expressed as a percentage of the total cell number.

### LDH assays

LDH assays were performed with the CytoTox 96 Non-Radioactive Cytotoxicity assay (Promega; G1780) following the manufacturer’s protocol. In brief, for each sample, the media was collected and cleared by centrifugation. For each sample, a total was calculated by re-suspending each corresponding well with 1× lysis buffer. The reaction was read at 492 nm using the Tecan Infinite M200 PRO plate reader (TECAN). Percentage LDH release = (sample-blank)/[(total-blank) + (sample-blank)] × 100. Assays were performed with at least four technical replicates.

### Temporal propidium iodide uptake assays

The media was replaced 6 h post-transfection with 10% FCS Opti-MEM supplemented with 1 μg/mL of propidium iodide (PI; Life Technologies; P3566) or alternatively, PI was added along with latrunculin A treatment. Additionally, a single well was lysed in 1% Triton, 10% FCS Opti-MEM supplemented with 1 μg/mL of PI to act as the reference total control and to set the gain limit. Plates were transferred to the Tecan Infinite M200 PRO plate reader (TECAN) set at 37°C and with a 5% CO_2_ atmosphere. Readings were set to and taken every 20 min for 24 h. Each condition was set up in quadruplicate. Data were normalized to individual relative fluorescence at time 0 h, and percentage PI uptake was calculated by dividing individual relative fluorescence units by the total and multiplying by 100.

### Flow cytometry

Transduced HeLa or J774A.1 were detached with warm trypsin-EDTA (Gibco), pelleted at 500 × *g,* and washed once in PBS. Pellets were gently re-suspended in Opti-MEM supplemented with 1 μg/mL PI. A positive control was generated by the resuspension of cells in 0.01% Triton X-100/PBS. Alternatively, a negative control was generated by resuspending cells in Opti-MEM alone. Cells were incubated for 15 min before recording 10,000 events per sample on the BD LSR Fortessa II Flow Cytometer (BD Biosciences). Data are presented as a percentage of PI-positive cells within the population.

### Generation of knockout bacterial strains

The deletion strain SL1344 Δ*spvB* was generated using the Lambda Red system, as described previously (62). Briefly, the primer set 13/14 was used to amplify the Kanamycin cassette from pKD4 with flanks targeting SL1334 *spvB*. Following electroporation, mutants were selected on LB agar with 25 μg/mL kanamycin (K1377) and re-streaked onto LB agar with 50 μg/mL of kanamycin. Colonies were screened by PCR using primer set 15/16. Successful clones were verified by sequencing with Eurofins.

### Infection

HeLa cells were seeded in 96 wp, 24 wp, or 6 wp at 2 × 10^4^, 1 × 10^5^, or 5 × 10^5^, respectively. HEK293A WT or KO was seeded in 96 wp, 24 wp, or 6 wp at 1.5 × 10^4^, 7.5 × 10^4^, or 3.75 × 10^5^, respectively. The bacterial strains *Salmonella enterica* Typhimurium SL1344 WT pACYC184-EV, Δ*spvB* pACYC184-EV, or Δ*spvB* pACYC184-*spvB* were cultured overnight in Luria broth (LB) supplemented with 10 μg/mL of chloramphenicol (C0378). Strains were sub-cultured by 1:33 dilution in LB for 3.5 h in the absence of antibiotics and added to cells at the multiplicity of infection (MOI) indicated. Plates were spun at 110 × *g* for 5 min before incubating for a further 10 min at 37°C and 5% CO_2_. Cell monolayers were washed twice in PBS, and medium was replaced with Opti-MEM with 10% FCS and 100 μg/mL of gentamicin (G1914) for 1 h. Where indicated, the MAP4K inhibitor PF was added at 40 μM. Following the 1-h incubation, plates for uptake assays were washed twice in PBS and lysed for 10 min in ice-cold 1% Triton X-100/PBS, diluted 10^1^–10^4^ in PBS, and plated on LB agar for CFU enumeration in quadruplicate. For infection efficiency assays, coverslips, in duplicate, were fixed in 3% paraformaldehyde (PFA)/PBS for 20 min, washed in PBS, and stored at 4°C before staining as described below. For replication, immunoblotting, and cell death assays, the media were replaced with Opti-MEM 10% FCS with 20 μg/Ll of gentamicin and incubated at 37°C and 5% CO_2_ for analysis at 20 and 24 h post-infection where stated.

### Replication assays

At 21 h post-infection, the bacteria in the media were pelleted by centrifugation at 400 *× g*. Cells were lysed in 1% Triton-X100/PBS, combined with the pelleted bacteria from the media, diluted in PBS, and plated on LB-agar for CFU enumeration in quadruplicate. Alternatively, to quantify intracellular or extracellular bacteria, the media were removed, and cell monolayers were washed twice in PBS. The media and washes were combined, diluted in PBS, and plated on LB agar for the enumeration of extracellular bacteria. Washed cell monolayers were lysed in 1% Triton-X100/PBS, diluted in PBS, and plated on LB-agar for enumeration of intracellular bacteria. Replication assays were performed with four technical replicates.

### Immunoblotting and antibodies

Detached cells were collected by centrifugation of the media at 6,000 *× g* for 3 min. Attached cells were lysed in ice-cold 1% Triton X-100/PBS supplemented with protease inhibitors (cOmplete; 04693159001) and phosphatase inhibitor (PhosSTOP; 4906837001), combined with the pelleted detached cells, and incubated for 15 min on ice with vortexing every 5 min. Lysates were cleared by centrifugation at 13,000 rpm (Eppendorf 5702R) for 20 min at 4°C, and supernatants were diluted in 4× loading dye (250 mM Tris [pH 6.8], 6% SDS, 40% glycerol, 1% bromophenol blue, and 0.8% β-mercaptoethanol) and boiled for 10 min. For infection, the insoluble fraction was resuspended in 2× loading dye directly and boiled for 10 min. Samples were separated by electrophoresis in a SDS-polyacrylamide gel in running buffer (20 mM Tris, 192 mM glycine, 1% [wt/vol] SDS) before transferring to an Immobilin-P PVDF membrane (IPVH00010) in transfer buffer (20 mM Tris-HCl, pH 8.3, 150 mM glycine) using the Turboblot system (BioRAD) at 15 V for 45 min. Membranes were blocked in 5% (wt/vol) dried milk (Marvel) or 5% (wt/vol) bovine serum albumin (BSA; A7906) in Tris-buffered saline (10 mM Tris, 150 mM NaCl), pH 7.4, with 0.1% (vol/vol) Tween-20 (TBS-T) for 60 min before incubating with the indicated primary antibody overnight at 4°C on a roller. Primary antibodies: rat monoclonal anti-GFP clone 3H9 (Chromotek; 3H9-100), mouse monoclonal anti-DnaK clone 8E2/2 (Enzo Life Sciences; ADI-SPA-880-F), anti-tubulin clone E7 (DSHB), anti-caspase-3 clone E-8 (Santa Cruz; sc-7272), anti-PARP-1 clone F2 (Santa Cruz; sc-8007), anti-AIF clone E1 (Santa Cruz; sc-13116), anti-EndoG clone B2 (Santa Cruz; 365359), anti-JNK clone D2 (Santa Cruz; sc-7345), rabbit monoclonal anti-phospho-SAPK/JNK (Thr183/Tyr185) clone 81E11 (Cell Signaling Technology; 4668), anti-cathepsin B clone D1C7Y XP (Cell Signaling Technology; 31718), rabbit polyclonal anti-caspase-3 (Enzo Life Sciences; ADI-AAS-103-D), and anti-HGK (Invitrogen; PA5-30579). Membranes were washed three times in TBS-T before incubating with secondary antibodies for 1.5 h. Secondary antibodies were HRP-conjugated goat anti-mouse IgG (Agilent Technologies Inc; P0447), goat anti-rabbit IgG (Agilent Technologies Inc; P0448), and goat anti-rat (Cell Signaling Technology; 7077) imaged using ECL Plus Western Blotting Substrate (Pierce; 32132X3) and the BioRAD imager system and Image Lab. Densitometry was calculated using ImageJ.

### Invasion efficiency assay

Fixed coverslips were incubated with 50 mM NH_4_Cl/PBS for 10 min before blocking in 1% BSA/PBS for 30 min. Coverslips were then stained with goat polyclonal anti-CSA-1 (KPL; 01-91-99) for 1 h, before washing three times in PBS and incubating with donkey anti-goat 488 (Invitrogen; A-11055) for 45 min. Coverslips were washed three times in PBS, and cells were permeabilized in 0.2% Triton-X100/PBS for 2.5 min and washed three times in PBS before repeating the primary and secondary staining again, albeit with the secondary anti-goat 555 (Invitrogen; A-21432). Coverslips were mounted using Aqua-Poly/Mount (Polysciences), and staining was imaged using the LSM 710 inverted confocal laser-scanning microscope (Zeiss GmbH). Bacteria that co-stained in the 488 and 555 channels were assigned as extracellular, whereas single 555-stained bacteria were assigned as intracellular. The percentage of cells with intracellular bacteria across two fields of view at 20× zoom (>100 cells) in duplicate per replicate was enumerated to calculate the uptake efficiency.

### Immunofluorescent staining, live imaging, and data acquisition

HeLa were transfected as described above or alternatively, transfected with 500 ng ptCMV-GFP-SpvB or 250 ng of pEGFP-N1 with 250 ng of pCDNA4/TO-EV and co-transfected with either 25 ng of KDEL-mCherry (ER lumen marker) or 40 ng of galectin-8-RFP. One hour before fixation, where indicated, Mitotracker (Invitrogen: M22426), Lysotracker Red (Invitrogen; L-7528), and/or Hoechst (ThermoFisher, 62,249) were added to the medium, following the manufacturer’s instructions. One hour later, detached cells were collected by centrifugation at 400 *× g*, resuspended gently in PBS, and mounted on a microslide with a coverslip. Cells were immediately imaged with the LSM 710 inverted confocal laser-scanning microscope (Zeiss GmbH). Attached cells were gently washed and fixed in 3% PFA/PBS for 20 min. Fixed coverslips were mounted and directly imaged or incubated with 50 mM NH_4_Cl/PBS, permeabilized, and blocked as above for further staining. Cells were stained with the following primary antibodies in 1% BSA/PBS for 2 h: rat anti-LAMP1 (DSHB; 1D4B). Coverslips were washed three times in PBS and incubated with the following secondary antibodies or stains in 1% BSA/PBS for 45 min: donkey anti-rat Alexa555 (Abcam; 150154), donkey anti-rabbit Alexa647 (Invitrogen; A-31573), Alexa555 phalloidin (ThermoFisher Scientific; A34055), and/or DAPI. Images were taken with the LSM 710 inverted confocal laser-scanning microscope (Zeiss GmbH) and analyzed with Zen 3.3 Blue edition (Zeiss GmbH). At least 25 cells from 5 fields of view at 63× zoom per condition were used for manual counts of nuclei, nuclear size, nuclear damage, and phalloidin measurements. The mean fluorescence intensity of phalloidin per GFP-positive cell was measured from 8 fields of view at 63× using the image analysis software Zen 3.3 Blue edition (Zeiss GmbH), with automatic background subtraction.

### Statistics

All experiments were carried out in triplicate and are representative of an average of at least three independent experiments unless stated otherwise. Data are the mean ± SD. Assays comparing two groups were analyzed by unpaired *t*-test with GraphPad Prism 9 Software, where *P* < 0.05 = *, *P* < 0.01 = **, *P* < 0.001 = ***, and *P* < 0.0001 = ****. Alternatively, data with multiple groups and one or two variables were analyzed using the one-way ANOVA or two-way ANOVA with Tukey’s post-test, respectively.

## RESULTS

### SpvB and latrunculin A cause cell death by inducing actin depolymerization

**Fig 1:**
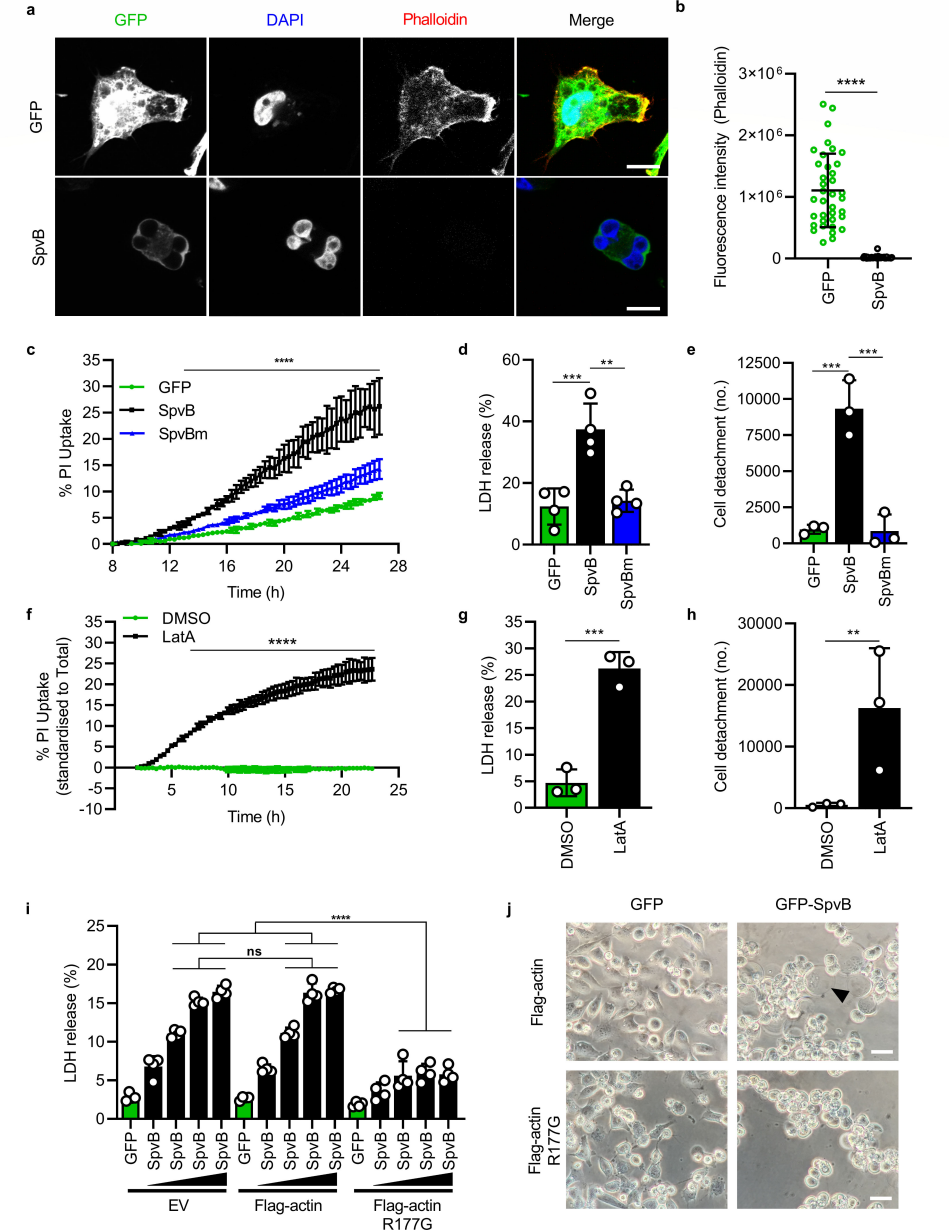
Actin ribosylation is an essential prerequisite for SpvB-dependent cell death. HeLa cells were transfected with pEGFP-N1 or ptCMV-GFP-SpvB and analyzed by confocal microscopy 24 h post-transfection. (**a**) Representative immunofluorescence staining of actin depolymerization. Cells were stained with phalloidin (polymerized actin; red) or DAPI (nuclei; blue) with GFP in green. Scale bar, 10 μm. (**b**) Mean fluorescence intensity of polymerized actin (red). Data are representative of three independent experiments. Statistical significance was analyzed by an unpaired Student’s *t*-test. *P* < 0.05 = *, *P* < 0.01 = **, *P* < 0.001 = ***, and *P* < 0.0001 = ****. (**c–e**) HeLa cells were transfected with pEGFP-N1, ptCMV-GFP-SpvB, or ptCMV-GFP-SpvBm as stated. (**c**) Temporal PI uptake relative to total (1% Triton treated). Representative of three independent experiments. (**d**) LDH release and (**e**) cell detachment 24 h post-transfection. Average of three independent experiments. Statistical significance was analyzed by one-way ANOVA with Tukey’s multiple comparison post-test. (**f–h**) HeLa cells were treated with DMSO control or 9.6 μM LatA. (**f**) Temporal PI uptake relative to total (1% Triton treated). Representative of three independent experiments. (**g**) LDH release and (**h**) cell detachment 18 h post-treatment. Average of three independent experiments. Statistical significance was analyzed by unpaired Student’s *t*-test. *P* < 0.05 = *, *P* < 0.01 = **, *P* < 0.001 = ***, and *P* < 0.0001 = ****. (**i and j**) HEK293-EV (EV), HEK293-Flag-actin, and HEK293-Flag-actin R177G cell lines were transfected with pEGFP-N1 or an increasing dose of ptCMV-GFP-SpvB (25, 50, 75, or 150 ng) and analyzed 24 h post-transfection. (**i**) LDH release. Representative of three independent experiments. Statistical significance was analyzed by two-way ANOVA with Tukey’s multiple comparison post-test. *P* < 0.05 = *, *P* < 0.01 = **, *P* < 0.001 = ***, and *P* < 0.0001 = ****. (**j**) Representative live light microscopy images showing typical cell morphology changes for each condition. Scale bar, 50 μm. Black arrow, vacuolized cell. Representative of three independent experiments.

Many previous studies have quantified cell death in response to mART actin depolymerization using measures of cytopathology, including cell rounding or cell detachment (46). Here, to investigate the mechanisms directly controlling actin depolymerization-induced cell death, rather than cell detachment alone, we set up assays to quantify cell death through membrane permeabilization (PI uptake) and membrane rupture (LDH release). To generate inducible cell lines, HeLa cells were first transduced to express SpvB or the previously described mART catalytically dead mutant of SpvB (SpvBm; E536D/E538D) with an N-terminal GFP-tag (28). As negative controls, cells were mock-transduced or transduced to express the STm effector SteE, which has no known action on the cytoskeleton (61). Twenty-four hours post-transduction, extensive actin depolymerization was detected following GFP-SpvB expression, as shown by the loss of phalloidin staining, and this was dependent upon its ADP-ribosyltransferase activity (Fig. S1a and b). Immunoblotting for GFP and tubulin as a loading control showed higher levels of GFP-SpvBm than GFP-SpvB (Fig. S1c). Therefore, different levels of SpvB did not explain the differences in phalloidin staining. Similarly, SpvB expression induced significantly higher mART-dependent PI uptake (Fig. S1d) and LDH release (Fig. S1e), in comparison to controls, indicating that mART activity causes cell death. We then attempted to make a doxycycline-inducible cell line for SpvB that we could clone out for high expression of SpvB in 100% of the population, but all cell lines tested died in the absence of induction. Due to the lack of an inducible cell line, along with potential effects of antiviral signaling from transduction, all further assays, where possible, were done using transient transfection. Transfection of HeLa cells and ectopic expression of GFP-SpvB induced significant actin depolymerization, cell detachment, and mART-dependent cell death (Fig. 1a through e; Fig. S2a). Furthermore, SpvB-dependent actin depolymerization and cell death were detected in multiple cell lines, including J774A.1 mouse macrophages (Fig. S3a and b and S4a and b), immortalized bone marrow–derived mouse macrophages, and MelJuso endothelial cells (Fig. S4c and d), indicating that cell death was not cell type-specific nor restricted to tumor-specific epithelial cells. Interestingly, treatment of HeLa cells with the actin depolymerizing alkaloid LatA induced robust PI uptake, LDH release, and cell detachment (Fig. 1f through h), indicating that cell death is a common consequence of actin depolymerizing agents and toxins. Although actin is the only known target of SpvB and LatA, ribosylation or binding of a secondary target that facilitates cell death is possible. SpvB specifically ADP-ribosylates actin at R177, and substitution at this site abolishes VahC and Photox-dependent ADP-ribosylation of actin while retaining the ability to hydrolyze ATP and polymerize (63). Therefore, to address whether SpvB-induced cell death is dependent upon actin ADP-ribosylation, we generated HEK293A cell lines constitutively expressing WT Flag-tagged actin (HEK-actin) or a Flag-tagged actin-R177G mutant (HEK-R177G). As a negative control, HEK293A cells were generated with an empty vector (HEK-EV). Both actin constructs were detected at a similar level (Fig. S2b). Following transfection and expression of GFP-SpvB, HEK-EV and HEK-actin cells underwent striking morphological changes, characterized by cell rounding, vacuolization, and cell death (Fig. 1i and j), in comparison to GFP-transfected controls. In contrast, while SpvB-expressing actin-R177G cell lines still underwent cell rounding, likely due to high levels of WT-actin depolymerization, SpvB-induced vacuolization, and cell death were significantly reduced even at high levels of SpvB expression (Fig. 1i; Fig. S2c). These results provide direct evidence for actin ribosylation as an essential step in SpvB-induced cell death.

### Caspases are dispensable for SpvB and latrunculin A-induced cell death

**Fig 2:**
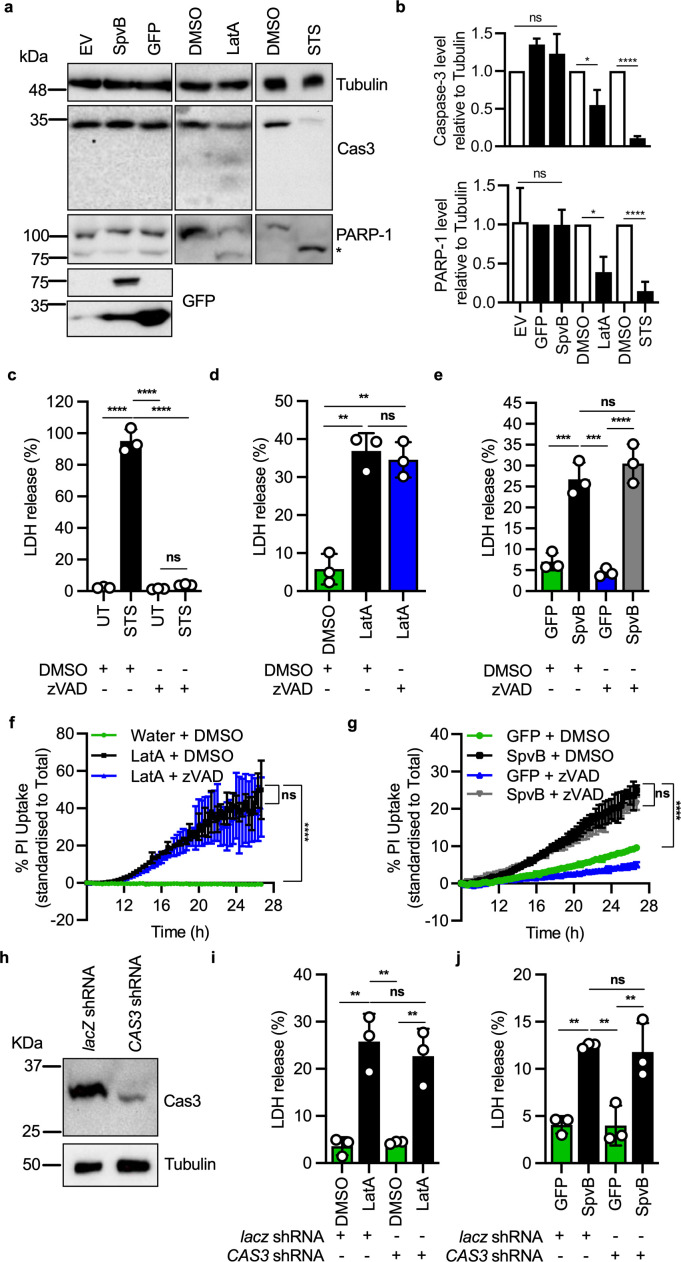
Caspase-3 activity is dispensable for SpvB- and latrunculin A-induced cell death. (**a and b**) HeLa cells were transfected with pEGFP-N1, ptCMV-GFP-SpvB, or empty vector (EV) or treated with DMSO (control) or 9.6 μM LatA or 1 μM STS. (**a**) At 24 h post-transfection or treatment, cell lysates were analyzed by SDS-PAGE and immunoblotting of caspase-3, PARP-1, tubulin, and GFP. (**b**) Average densitometry for (**a**) procaspase-3 and full-length PARP-1, relative to tubulin. GFP was immunoblotted to control for expression. *, cleaved PARP-1. Average of three independent experiments. Statistical significance was analyzed by unpaired Student’s *t*-test. *P* < 0.05 = *, *P* < 0.01 = **, *P* < 0.001 = ***, and *P* < 0.0001 = ****. (**c**) HeLa cells were treated with 1 μM STS with or without 50 μM z-VAD-FMK and analyzed by an LDH release assay 18 h post-treatment. Representative of two independent repeats. (**d**) HeLa cells were treated with DMSO (control) or 9.6 μM latrunculin A with or without the addition of 50 μM z-VAD-FMK. At 18 h post-treatment, cell death was measured by an LDH release assay. Average of three independent experiments. Average of three independent experiments. (**e**) HeLa cells were transfected with pEGFP-N1 or ptCMV-GFP-SpvB with or without the addition of 50 μM z-VAD-FMK at 6 h post-transfection. At 24 h post-transfection, cell death was measured by an LDH release assay. Average of three independent experiments. (**f and g**) Cells were treated as in panels **d** and **e**, and temporal PI uptake relative to total (1% Triton treated) was measured. Representative of three independent experiments. (**h–j**) HeLa cells were transduced to express YFP-tagged miR30E shRNA targeting *CASP3* or a non-targeting (*lacZ*) control. (**h**) Knockdown efficiency was determined by SDS-PAGE and immunoblotting of cell lysates with antibodies for caspase-3 and tubulin. (**i and j**) HeLa Cas3-shRNA and HeLa LacZ-shRNA cell lines were (**i**) treated with LatA or DMSO control or (**j**) transfected with pEGFP-N1 or ptCMV-GFP-SpvB, and cell death was analyzed by an LDH release assay at 18 h post-treatment and 24 h post-transfection. Average of three independent repeats. Statistical significance was analyzed by two-way ANOVA with Tukey’s multiple comparison post-test. *P* < 0.05 = *, *P* < 0.01 = **, *P* < 0.001 = ***, and *P* < 0.0001 = ****.

It has been proposed that actin depolymerization-induced cell death is mediated through mART activation of caspase-3, leading to caspase-dependent apoptosis (26, 27). To investigate a role for caspases in SpvB- and LatA-induced cell death, we transfected HeLa cells with a vector expressing SpvB or treated cells with LatA and immunoblotted lysates for caspase-3 and its substrate PARP-1. Staurosporine A (STS) was used as a positive control for caspase activation and caspase-dependent apoptosis. STS induced strong activation of caspase-3 as demonstrated by complete loss of the procaspase-3 and significant cleavage of PARP-1 (Fig. 2a and b). LatA induced cleavage of PARP-1 and partial loss of procaspase-3; however, this was substantially reduced in comparison to STS treatment (Fig. 2a and b). In comparison to GFP, no PARP-1 cleavage nor changes in procaspase-3 levels were detected following the expression of GFP-SpvB, despite significant LDH release (Fig. 2a, b and e). Partial PARP-1 cleavage was detected following all transfection conditions, including the EV alone control, and therefore this is likely to represent a response to the transfection reagent/protocol itself. It was possible that SpvB did cause caspase activation, but that this was below the limit of our detection. Therefore, to further investigate the role of caspases in SpvB-induced cell death, the cell death assays were repeated with the addition of the pan-caspase inhibitor zVAD-FMK. Despite complete rescue of STS-induced apoptosis and LDH release (Fig. 2c), zVAD-FMK treatment provided no protection to SpvB-expressing or LatA-treated cells (Fig. 2d through g), implying that cell death from SpvB is caspase-independent. To test this further, we generated stable caspase-3-targeting shRNA-expressing cell lines or cell lines targeting *lacZ* as a negative control. Although efficient knockdown (KD) of caspase-3 was confirmed by Western blot (Fig. 2h), no difference was detected for SpvB- or LatA-induced cell death when comparing LacZ and caspase-3 KD-treated or transfected cell lines (Fig. 2i and j; Fig. S5). This is consistent with our findings that pan-caspase inhibition failed to rescue cell death driven by SpvB and LatA. Together, the data show that caspases are dispensable for actin depolymerization-induced cell death. This raised the question: what are the cellular signaling pathway(s) and executioner(s) responsible for cell death induced by actin depolymerization?

### Actin depolymerization-induced cell death is morphologically distinct from defined cell death modalities

**Fig 3:**
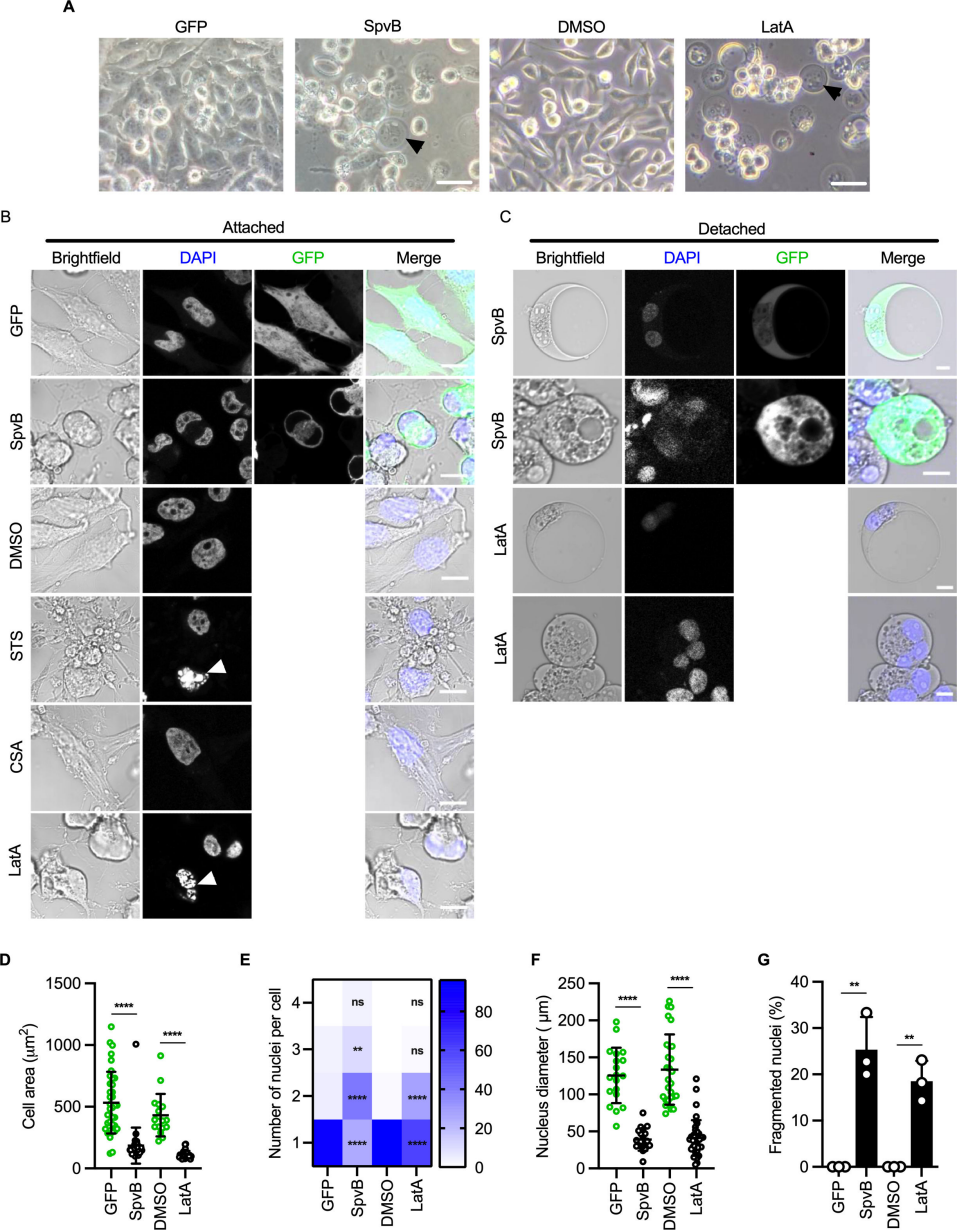
SpvB and latrunculin A induce cytoplasmic vacuolization and nuclear shrinkage, condensation, and fragmentation. HeLa cells were transfected with pEGFP-N1 or ptCMV-GFP-SpvB or treated with DMSO or 9.6 μM LatA and analyzed 24 h post-transfection and 18 h post-treatment, respectively. (**a**) Representative live light microscopy images showing typical cell morphology changes. Scale bar, 50 μm. Black arrow, vacuolized bubble-like cells; white arrows, round shrunken cells. (**b–g**) HeLa cells were transfected with pEGFP-N1 or ptCMV-GFP-SpvB or treated with DMSO, 1 μM STS, 40 μM CSA, or 9.6 μM LatA, where indicated. (**b and c**) Representative confocal immunofluorescence images of (**b**) fixed attached cells or (**c**) live detached cells, 24 h post-transfection or 18 h post-treatment. Cells were stained with DAPI 1 h prior to fixation or imaging. Green, GFP; Blue, DAPI (nuclei). Scale bar, 10 μm. White arrows, damaged nuclei. (**d**) Cell area of attached cells. Statistical significance was analyzed by unpaired Student’s *t*-test. (**e**) Number of nuclei per attached cell. Statistics shown for GFP vs SpvB and DMSO vs LatA. Statistical significance was analyzed by two-way ANOVA with Tukey’s multiple comparison post-test. (**f**) Average diameter of nuclei in attached cells. (**g**) Percentage of fragmented nuclei in attached cells. Representative of three independent experiments. Statistical significance was analyzed by unpaired Student’s *t*-test. *P* < 0.05 = *, *P* < 0.01 = **, *P* < 0.001 = ***, and *P* < 0.0001 = ****.

The type and mode of cell death can be classified by the distinct morphological changes that occur leading up to death (64). For example, caspase-dependent apoptosis induces cell shrinkage, blebbing, and apoptotic body formation (64). In contrast, necrosis induces cell swelling and membrane rupture, whereas paraptosis induces cytoplasmic vacuolization through swelling of the ER (64). Therefore, to establish the type of cell death induced by actin depolymerization, we documented the cellular and morphological changes following ectopic expression of SpvB or LatA treatment by light and confocal microscopy (Fig. 3a through c). As controls, cells were treated with STS to induce apoptosis or cyclosporine A (CSA) to induce paraptosis (65, 66). As expected, STS induced cell shrinkage, blebbing, and DNA condensation and fragmentation, whereas CSA induced vacuolization with minimal changes to cell shape or size (Fig. 3b). In striking contrast, SpvB and LatA both induced two distinct morphological changes. The first was characterized by cell rounding, shrinkage, and cytoplasmic vacuolization with multinucleation, nuclear shrinkage, and nuclear fragmentation of attached cells (Fig. 3a, b, and d through g; Fig. S6a through c; white arrows). Alternatively, cells detached and swelled up forming a single large vacuole or “bubble-like” morphology, as indicated by black arrows (Fig. 3a and c). Furthermore, these changes were mART-dependent, indicating they occur in response to actin depolymerization (Fig. S6a through c). DNA damage is consistent with previous findings (26, 27), while multinucleation has been previously reported for SpvB and is consistent with G2/M cell cycle inhibition (27). However, although SpvB-induced vacuolization of cells can be seen in images of previous publications (26, 27), this was not reported or investigated by the authors. Two major types of cell death induce vacuolization: paraptosis and methuosis (64). Paraptosis-induced vacuoles are derived from the ER and mitochondria, whereas methuosis-induced vacuoles are derived from macropinosomes. Since actin polymerization is essential for macropinocytosis and inhibited by latrunculin treatment (67), methuosis was not examined further. To determine whether SpvB-induced vacuoles are derived from the ER and/or mitochondria, cells were transfected to express a luminal ER marker tagged with mCherry or stained with Mitotracker and co-transfected with a vector expressing GFP-SpvB or treated with LatA and analyzed by confocal microscopy. CSA was used as a positive control, with vacuoles staining positive for the luminal ER marker (Fig. S7a). However, SpvB- and LatA-induced vacuoles did not co-localize with the ER marker or Mitotracker (Fig. S7a and b). Furthermore, unlike STS treatment, Mitotracker staining was not diffuse, implying the mitochondria remain intact during SpvB- and LatA-induced cell death (Fig. S7b). Taken together, the morphological changes driven by actin depolymerizing agents share the common features of cell rounding and vacuolization along with multinucleation, nuclear shrinkage, and DNA damage. Therefore, they do not correspond with any previously described cell death pathway. Notably, the “bubble-like” morphology (Fig. 1j; Fig. 3a and c) has not been previously described or associated with any mode of cell death. Given these atypical morphologies, it was not possible to identify the type of cell death using morphology alone. Therefore, we next explored potential pro-cell death signaling pathways reported to be activated by actin depolymerizing agents and toxins.

### SpvB-induced cell death is MAP4K/JNK-dependent

**Fig 4:**
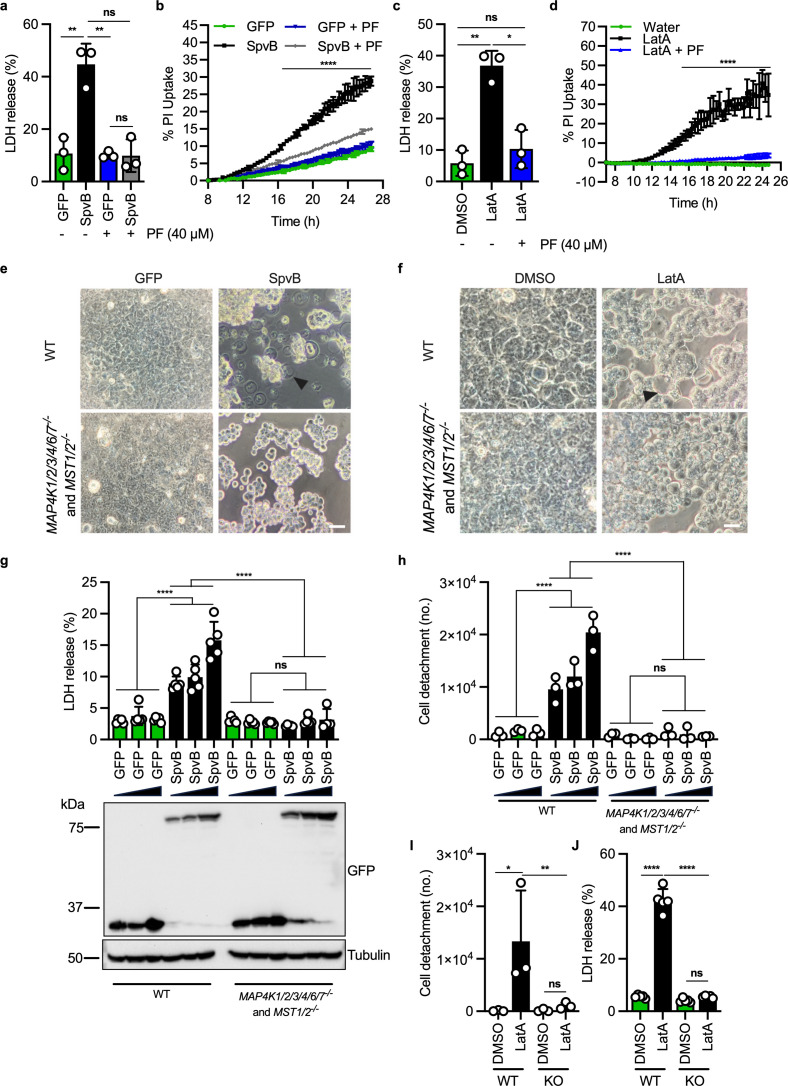
The MAP4K family drives cell death induced by excessive actin depolymerization. (**a and b**) HeLa cells were transfected with pEGFP-N1 or ptCMV-GFP-SpvB with or without the addition of 40 μM PF-06260933 (PF) at 6 h post-transfection. Cell death was measured by (**a**) LDH release at 24 h post-transfection or by (**b**) a temporal PI uptake assay. (**c and d**) HeLa cells were treated with DMSO or 9.6 μM LatA with or without the addition of 40 μM PF. Cell death was measured by (**c**) LDH release at 24 h post-transfection or by (**d**) a temporal PI uptake assay. (**e–j**) HEK293A or HEK293A KO (*MAP4K1/2/3/4/6/7*^−/−^ and *MST1/2*^−/−^) cells were transfected with an increasing dose of pEGFP-N1 or ptCMV-GFP-SpvB, or treated with DMSO or 9.6 μM LatA and analyzed 24 h post-transfection or 18 h post-treatment, respectively. (**e and f**) Live light microscopy images showing typical cell morphology changes. Scale bar, 20 μm and 10 μm, respectively. Black arrows, vacuolized bubble-like cells. (**g**) LDH release 24 h post-transfection. Representative of three independent experiments. In parallel, cell lysates were analyzed by SDS-PAGE and immunoblotting for relative protein expression using antibodies against GFP and tubulin. Representative of three independent experiments. (**h**) Cell detachment at 24 h post-transfection. Average of three independent experiments. (**i**) Cell detachment and (**j**) LDH release at 18 h post-treatment with LatA or DMSO. Representative of three independent experiments. Statistical significance was analyzed by two-way ANOVA with Tukey’s multiple comparison post-test. *P* < 0.05 = *, *P* < 0.01 = **, *P* < 0.001 = ***, and *P* < 0.0001 = ****.

Depolymerization of the actin cytoskeleton results in the modulation of several cellular signaling pathways, including those controlling cell cycle (2), autophagy (68), and MAP4K-dependent Hippo signaling (69). Interestingly, MAP4Ks have been investigated extensively as inducers of cardiomyocyte and motor neuron (MN) cell death (70, 71), with cell death initiated through downstream activation of the stress kinase JNK (70). To investigate whether cell death induced by actin depolymerization is MAP4K-dependent, we transfected HeLa cells to express GFP-SpvB or treated cells with LatA and further treated cells with the MAP4K4/6/7-specific inhibitor PF-6260933 (PF) or vehicle control and analyzed cell death by LDH release and PI uptake. The addition of PF abolished SpvB (Fig. 4a and b) and LatA-induced cell death (Fig. 4c and d), with controls showing no effect of PF on SpvB levels (Fig. S8a). Surprisingly, whereas PF rescued LatA-induced cell death, it did not prevent caspase-dependent PARP-1 cleavage. Alternatively, zVAD rescued PARP-1 cleavage but had no effect on cell death (Fig. S8b and c). This is consistent with our finding that cell death induced by actin depolymerization is caspase-independent (Fig. 2). Of the MAP4Ks, MAP4K4 is the most widely reported to regulate cell death (70–72). Therefore, to determine whether MAP4K4 induces cell death in response to SpvB and LatA, we generated a HeLa MAP4K4-deficient cell line. Despite efficient MAP4K4 knockdown (Fig. S9a), only partial rescue of SpvB-induced vacuolization and SpvB- or LatA-induced LDH release was detected (Fig. S9b through d). This might have been due to redundancy among the MAP4K family and would support previous findings of redundancy in the MAP4Ks activating Hippo signaling in response to LatA treatment (69) or stress-induced JNK activation and cell death in MN (73). Therefore, to further test whether cell death is MAP4K-dependent, we transfected HEK293A wild type (WT) or the previously generated HEK293A KO (*MAP4K1/2/3/4/6/7^−/−^* and *MST1/2^−/−^*) (69) cell line, which lacks all Hippo kinases (MAP4K and MST1/2), with a SpvB-expressing vector, or treated cells with LatA and analyzed the morphological changes and cell death. As with HeLa cells, ectopic expression of SpvB or LatA treatment of HEK293A WT cells induced cell rounding, detachment, vacuolization, and bubble-like morphological changes (Fig. 4e and f). In contrast, although the HEK293A KO (*MAP4K1/2/3/4/6/7^−/−^* and *MST1/2^−/−^*) cells rounded up (suggesting actin depolymerization), there was a complete absence of the “bubble-like” cell formation associated with actin depolymerization-induced cell death (Fig. 4e and f). Crucially, cell death and detachment were rescued by the lack of MAP4K1/2/3/4/6/7 and MST1/2, despite high levels of SpvB expression (Fig. 4g through j). Overall, these data show that the MAP4K family has an essential role in driving cell death in response to actin depolymerization.

**Fig 5:**
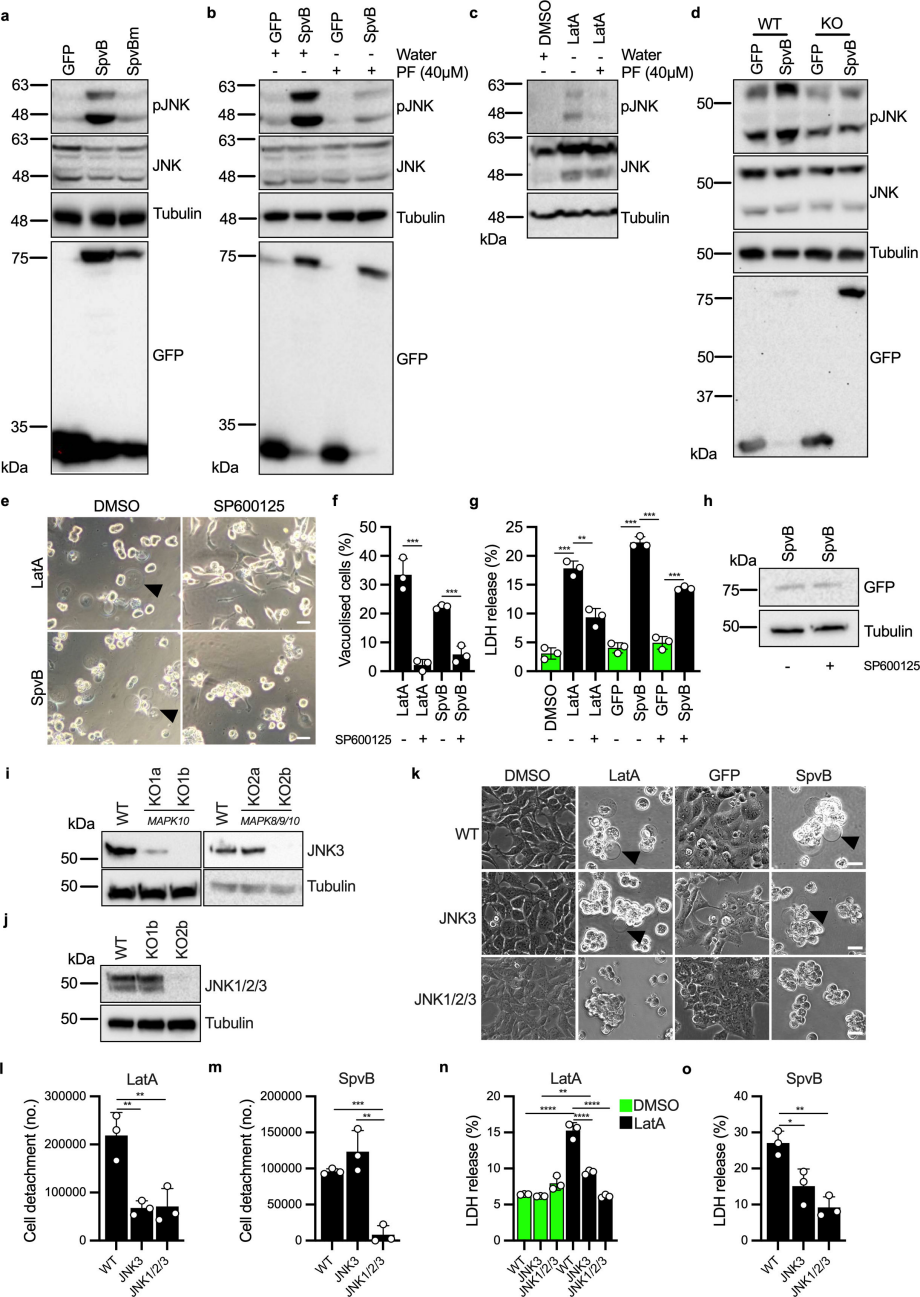
MAP4K-dependent cell death induced by actin depolymerization signals via JNK. (**a**) Representative immunoblots for JNK, phospho-JNK Thr183/Tyr185, GFP, and tubulin following transfection of HeLa cells with pEGFP-N1, ptCMV-GFP-SpvB, or ptCMV-GFP-SpvBm (catalytic dead). Representative of three independent experiments. (**b and c**) Representative immunoblots for JNK, phospho-JNK Thr183/Tyr185, GFP, and tubulin following (**b**) HeLa cell transfection with pEGFP-N1 or ptCMV-GFP-SpvB with or without 40 μM PF-06260933 (PF) or (**c**) treatment of HeLa cells with DMSO or 9.6 μM LatA with or without 40 μM PF at 24 h post-transfection or 18 h post-treatment, respectively. Representative of three independent experiments. (**d**) Representative immunoblots for JNK, phosphorylated JNK, GFP, and tubulin following transfection of HEK293A and HEK293 KO (*MAP4K1/2/3/4/6/7*^−/−^ and *MST1/2*^−/−^) cells with pEGFP-N1 or ptCMV-GFP-SpvB. Representative of two independent experiments. (**e–h**) HeLa cells were treated with DMSO or 9.6 μM LatA or transfected with pEGFP-N1 or ptCMV-GFP-SpvB and treated 6 h later with or without SP600125. (**e**) Representative live light microscopy image of cell morphology. Scale bar, 25 μm. Black arrows, vacuolized bubble-like cells. (**f**) Number of detached vacuolized cells. Statistical significance analyzed by unpaired Student’s *t*-test. (**g**) LDH release. Representative of three independent experiments. Statistical significance analyzed by two-way ANOVA with Tukey’s multiple comparison post-test. (**h**) In parallel, cell lysates were analyzed by SDS-PAGE and immunoblotting with anti-GFP and anti-tubulin antibodies to determine relative protein expression. (**i and j**) Knockouts of JNK3 (*MAPK10;* KO1) and JNK1/2/3 (*MAPK8/9/10;* KO2) were generated by transfection of HEK293A with pX330 with guides specific to *MAPK8, MAPK9,* and *MAPK10*. Cell lysates from the potential knockout clones were analyzed by SDS-PAGE and immunoblotting with anti-JNK3 and anti-tubulin antibodies. (**j**) Cell lysates from clones lacking JNK3 were then analyzed by SDS-PAGE and immunoblotting with a pan anti-JNK1/2/3 antibody and anti-tubulin. (**k–o**) HEK293A KO cell lines JNK3 (*MAPK8^−/−^;* KO1) and JNK1/2/3 (*MAPK8/9/10^−/−^; KO2*) were treated with DMSO or 9.6 μM LatA or transfected with pEGFP-N1 or ptCMV-GFP-SpvB and analyzed at 18 h post-treatment or 24 h post-transfection, respectively. (**k**) Representative live light microscopy image of cell morphology. Scale bar, 100 μm. Black arrows, vacuolized bubble-like cells. (**l and m**) Cell detachment. Statistical significance analyzed by unpaired Student’s *t*-test. (**n and o**) LDH release. Statistical significance analyzed by (**n**) two-way ANOVA or (**o**) one-way ANOVA with Tukey’s multiple comparison post-test. Representative of three independent experiments. *P* < 0.05 = *, *P* < 0.01 = **, *P* < 0.001 = ***, and *P* < 0.0001 = ****.

Given MAP4K-induced cell death in MNs is dependent on the stress kinase JNK, we asked whether JNK facilitates cell death downstream of MAP4Ks in response to actin depolymerization (73). First, the phosphorylation status of JNK was investigated by immunoblotting of lysates. Expression of SpvB, in a mART-dependent manner, and LatA treatment of cells, induced strong phosphorylation of JNK at Thr183/Tyr185 in comparison to GFP or DMSO controls (Fig. 5a through c). Furthermore, the addition of PF abolished SpvB- and LatA-induced phosphorylation of JNK (Fig. 5b and c). Similarly, phosphorylation was reduced in SpvB-transfected HEK293A *MAP4K1/2/3/4/6/7^−/−^* and *MST1/2^−/−^* cells in comparison to WT cells (Fig. 5d). Together, our data show that this event is downstream of MAP4K activation. To investigate the potential role of JNK in SpvB/LatA-induced cell death, we repeated the cell death assays in the presence or absence of the JNK inhibitor SP600125. As seen with MAP4K inhibition, the addition of SP600125 prevented vacuolization and significantly reduced LDH release in response to SpvB and LatA (Fig. 5e through h). SP600125 is reported to target all three isoforms of JNK, including JNK1 (MAPK8), JNK2 (MAPK9), and JNK3 (MAPK10), and is also reported to have off-target effects, including CDK2 (74). Therefore, to further explore the potential role of JNK in MAP4K4-dependent cell death, we generated single JNK3 KO (*MAPK10*^−/−^; KO1) and triple JNK JNK1/2/3 KO (*MAPK8/9/10^−/−^*; KO2) HEK293A cell lines. First, JNK3 KO in single and triple KO cell lines was confirmed by immunoblotting with a JNK3-specific antibody, with clone KO1b for the single JNK3 KO and clone KO2b for the triple KO transfections each lacking a band corresponding to JNK3 (Fig. 5i). Clones for single JNK3 KO (KO1b) and triple JNK KO (KO2b) were then analyzed with an antibody that recognizes all three isoforms (Fig. 5j), verifying generation of a JNK3-specific knockout (KO2b) and a cell line lacking all three JNKs (KO1b). As seen with MAP4K4 KO, Triple JNK1/2/3 KO (KO2b; *MAPK8/9/10^−/^*^−^) completely rescued cell detachment, vacuolization, and cell death (LDH release) in response to LatA treatment and SpvB expression (Fig. 5k through o). MAP4K4-dependent cell death is reported to be JNK3-specific; however, although we detected significant reduction in LDH release from LatA-treated and SpvB-expressing JNK3 KO cells in comparison to the WT cells, the rescue was only partial (Fig. 5n and o). Furthermore, the single JNK3 KO had no impact on cell detachment in response to SpvB expression (Fig. 5m), suggesting that all three JNK isoforms work in concert to drive actin-depolymerization-induced MAP4K4-dependent cell death.

### SpvB and latrunculin A-induced death is partially rescued through cathepsin inhibition

Cell death in response to JNK phosphorylation is reported to proceed via multiple caspase-independent pathways due to varying stimuli and their concentrations or the cell lines used. This includes mitochondrion-dependent pathways through the release of endonuclease G (ENDOG) or apoptosis-inducing factor (AIF) that induce DNA damage or through mitochondrial-independent pathways proceeding through lysosomal permeabilization, or rupture, and cathepsin release, or finally through induction of necrosis/necroptosis (75–78).

**Fig 6:**
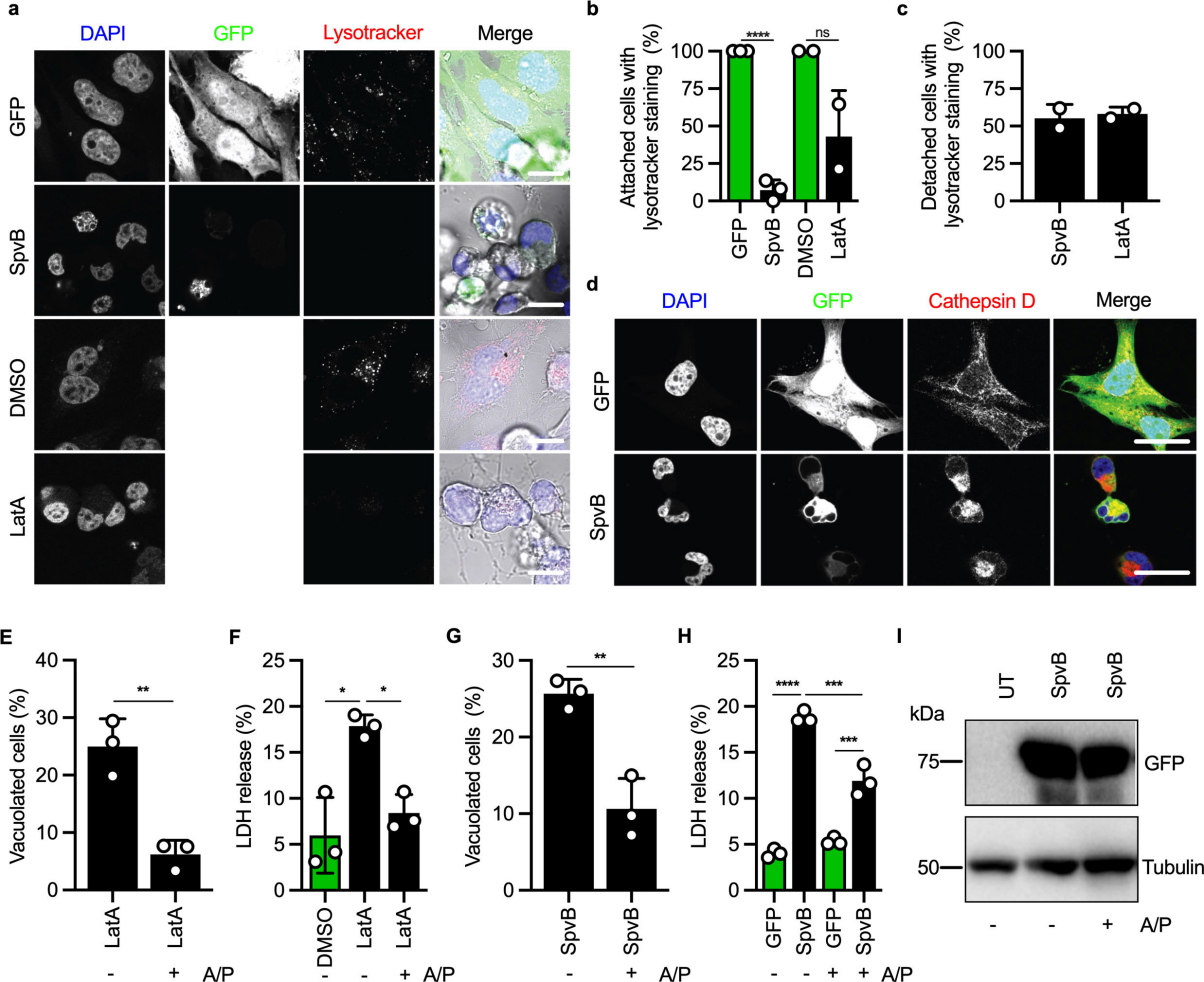
Actin depolymerization induces LMP and cathepsin-mediated cell death. (**a–d**) HeLa cells were transfected with pEGFP-N1 or ptCMV-GFP-SpvB or treated with 9.6 μM LatA or DMSO control, where stated, and analyzed 24 h post-transfection and 18 h post-treatment, respectively. (**a**) Representative confocal immunofluorescence staining of lysosomes. Cells were stained with Lysotracker (red) or DAPI (nuclei; blue) with GFP in green. Scale bar, 10 μm. (**b and c**) Enumeration of Lysotracker positive (**b**) attached and (**c**) detached cells. Representative of three independent experiments. Statistical significance was analyzed by unpaired Student’s *t*-test. *P* < 0.05 = *, *P* < 0.01 = **, *P* < 0.001 = ***, and *P* < 0.0001 = ****. (**d**) Representative immunofluorescence staining of LAMP1-positive vesicles. Cells were stained with anti-LAMP1 (red) or DAPI (nuclei; blue) with GFP in green. Scale bar, 20 μm. Representative of three independent experiments. (**e and f**) HeLa cells were treated with 9.6 μM LatA or DMSO control with or without 30 μM ALLM and 15 μM pepstatin A (A/P) and analyzed 18 h post-treatment. (**e**) Number of detached vacuolized cells. Statistical significance was analyzed by unpaired Student’s *t*-test. (**f**) LDH release. Average of three independent experiments. Statistical significance was analyzed by two-way ANOVA with Tukey’s multiple comparison post-test. (**g–i**) HeLa cells were transfected with pEGFP-N1 or ptCMV-GFP-SpvB with or without 30 μM ALLM and 15 μM pepstatin A (A/P) and analyzed 24 h post-transfection. (**g**) Number of detached vacuolized cells. Statistical significance was analyzed by unpaired Student’s *t*-test. (**h**) LDH release. Average of three independent experiments. Statistical significance was analyzed by two-way ANOVA with Tukey’s multiple comparison post-test. (**i**) In parallel, cell lysates were analyzed by SDS-PAGE and immunoblotted for SpvB expression using anti-GFP and anti-tubulin antibodies. Representative of three independent experiments. *P* < 0.05 = *, *P* < 0.01 = **, *P* < 0.001 = ***, and *P* < 0.0001 = ****. ns, non-significant.

To investigate the potential effectors of cell death downstream of MAP4K/JNK, we initially generated stable *AIF* and *ENDOG* targeting shRNA-expressing HeLa cell lines. Despite efficient knockdown, as confirmed by immunoblotting (Fig. S10a and b), no significant effect on LDH release in response to SpvB expression or LatA treatment was observed (Fig. S1oc through h). Of note, following SpvB expression, AIF does not enter the nucleus (Fig. S1oi). Lack of AIF release from the mitochondria is consistent with our findings that the mitochondrial membrane remains intact following SpvB transfection (Fig. S7b). Taken together, this suggests that cell death is unlikely to proceed via pathways dependent upon mitochondrial depolarization. Next, we examined the endo-lysosomal compartment by immunofluorescent staining of GFP or GFP-SpvB-expressing or LatA-treated cells. Cells were stained with Lysotracker (a lysosomal acidification marker) or LAMP1 to monitor the acidity and appearance of the lysosomal compartment. Lysotracker staining revealed clear cytoplasmic puncta in the GFP-expressing and DMSO control-treated cells (Fig. 6a). Conversely, SpvB expression or LatA treatment abolished lysotracker staining in most cells, indicating disruption of lysosomal maturation and/or lysosomal permeabilization (79) (Fig. 6a through c). Furthermore, LAMP1 in SpvB-expressing cells localized in larger, more intense aggregates in comparison to GFP-expressing cells, consistent with lysosomal disruption (Fig. 6d). However, this could also be attributed to perinuclear clustering or the smaller cytoplasmic volume of SpvB-expressing cells in comparison to controls. Given that lysosome acidification is required for cathepsin processing, we immunoblotted for cathepsin B and D. Immunoblotting revealed that bands corresponding to active, mature cathepsin D and B following LatA treatment, or cathepsin B alone following SpvB expression, were reduced (Fig. S11). Taken together, these data suggest disruption of the endo-lysosomal compartment following actin depolymerization. Lysosomal swelling and pH neutralization, resulting in a lack of Lysotracker staining, are key features of lysosomal membrane permeabilization, resulting in the release of cathepsins into the cytoplasm, which trigger apoptosis and/or necrosis of the cell (79). To explore cathepsins as potential effectors of actin depolymerization-induced cell death, we treated SpvB-expressing or LatA-treated cells with a cocktail of cathepsin inhibitors (ALLM/pepstatin A) to broadly inhibit the activity of the major cathepsins, including cathepsin B, D, and L. Co-treatment with pepstatin A and ALLM significantly reduced vacuolization and LDH release with no effect on SpvB expression (Fig. 6e and f), suggesting that cathepsins contribute as downstream executioners of cell death in response to actin depolymerization.

### SpvB induces MAP4K-dependent epithelial cell death after delivery from *Salmonella*

**Fig 7:**
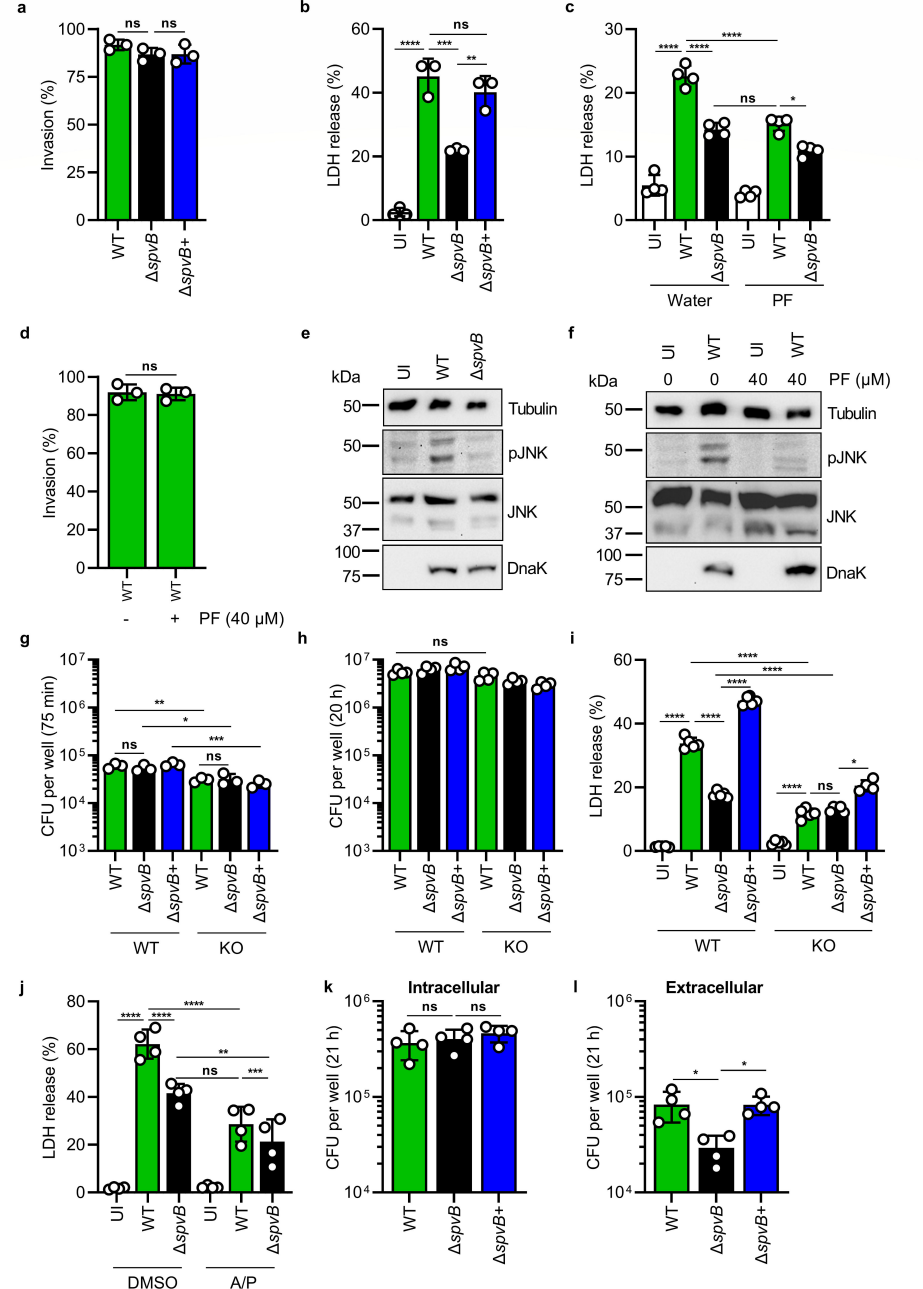
*Salmonella* infection induces SpvB-dependent MAP4K/cathepsin-dependent epithelial cell death. HeLa cells were infected with *Salmonella* SL1334 strains WT pACYC-EV, Δ*spvB* pACYC-EV, or Δ*spvB* pACYC-*spvB* at an MOI of 25. (**a**) Cells were fixed at 75 min p.i., and invasion efficiency (percentage of cells with intracellular staining for *Salmonella*) was enumerated by inside–outside immunofluorescence staining for *Salmonella* with an anti-CSA antibody. (**b**) LDH release assay at 21 h post-infection (p.i.). Average of three independent experiments. Statistical significance was analyzed using one-way ANOVA with Tukey’s multiple comparison post-test. (**c and d**) HeLa cells were infected with WT *Salmonella* SL1334 pACYC-EV or Δ*spvB* pACYC-EV at an MOI of 25 with or without the addition of 40 μM PF-06260933 (PF) at 15 min p.i. (**c**) Cell death was analyzed by an LDH release assay at 21 h post-infection. Representative of at least three independent experiments. Statistical significance was analyzed by two-way ANOVA with Tukey’s multiple comparison post-test. (**d**) In parallel, cells were fixed at 75 min p.i., and invasion efficiency (percentage of cells with intracellular staining for *Salmonella*) was enumerated by inside-outside immunofluorescence staining for *Salmonella* with an anti-CSA antibody. Average of three independent experiments. Statistical significance was analyzed by unpaired Student’s *t*-test. (**e**) Representative immunoblots for JNK, phospho-JNK (Thr183/Tyr185), tubulin, and DnaK at 21 h post-infection. Representative of three independent experiments. (**f**) HeLa cells were infected with WT *Salmonella* SL1334 pACYC-EV at an MOI of 25 with or without the addition of 40 μM PF-06260933 (PF) and analyzed at 24 h p.i. Representative immunoblots for JNK, phospho-JNK (Thr183/Tyr185), tubulin, and DnaK at 21 h post-infection. Representative of three independent experiments. (**g–i**) HEK293A or HEK293A KO (*MAP4K1/2/3/4/6/7^–/–^* and *MST1/2^–/–^*) cells were infected with *Salmonella* SL1334 strains WT pACYC-EV, Δ*spvB* pACYC-EV, or Δ*spvB* pACYC-*spvB* at an MOI of 50. (**g**) Invasion was calculated by lysing cells at 75 min p.i. and enumerating colony-forming units (CFU) per well on LB agar. (**h**) Bacterial numbers at 20 h p.i. were calculated by combining bacteria pelleted from the media with whole-cell lysates and enumerating CFU per well on LB agar. (**i**) LDH release assay at 20 h post-infection. Statistical significance was analyzed by two-way ANOVA with Tukey’s multiple comparison post-test. (**j**) HeLa cells were infected as in panel **a** with the addition of 30 μM ALLM and 15 μM pepstatin A (A/P) or DMSO. (**k and l**) Number of intracellular (**k**) or extracellular (**l**) bacteria was enumerated by plating the whole-cell lysate or media on LB agar following infection of HeLa cells with WT *Salmonella* SL1334 pACYC-EV or Δ*spvB* pACYC-EV at an MOI of 25 for 21 h. Representative of three independent experiments. Statistical significance was analyzed using one-way ANOVA with Tukey’s multiple comparison

We next investigated whether the MAP4K/JNK/cathepsin cell death pathway we identified using ectopic expression of SpvB occurs during STm infection following translocation of endogenous SpvB. To explore the role of SpvB during infection, we generated a *spvB* knockout in the SL1344 strain of STm and infected HeLa cells with SL1344 WT, an isogenic deletion strain (Δ*spvB*) or the complemented strain (Δ*spvB* pACYC-*spvB;* Δ*spvB+*) and analyzed cell death and signaling 21 h post-invasion. As a control, the invasion efficiency of each strain was quantified by immunofluorescence microscopy (Fig. 7a). Despite similar levels of invasion, WT STm infection induced significantly more LDH release than infection with Δ*spvB* (Fig. 7a and b). Importantly, complementation through constitutive expression of SpvB rescued the amount of LDH released to that of the WT strain (Fig. 7b), indicating that cell death during infection is partially SpvB-dependent. To investigate the potential role of MAP4Ks in this cell death pathway, the infections were repeated in the presence of MAP4K4/6/7 inhibitor PF. The addition of PF significantly reduced LDH release following WT STm infection, reducing LDH release levels to that of the Δ*spvB*-infected cells, with no effect on cell invasion (Fig. 7c and d). Moreover, JNK phosphorylation followed a similar pattern, where infection with the Δ*spvB* strain or treatment of WT STm-infected cells with PF reduced STm-dependent JNK phosphorylation (Fig. 7e and f). Taken together, these data indicate that SpvB drives JNK phosphorylation and cell death during infection in a MAP4K-dependent manner. As an alternative approach to investigate a role for the MAP4K family in cell death caused by STm and SpvB, the infections with the WT, Δ*spvB,* or Δ*spvB*+ strains were repeated using the HEK293A WT and HEK293A *MAP4K1/2/3/4/6/7^–/–^* and *MST1/2^–/–^* cells. Loss of MAP4K1/2/3/4/6/7 and MST1/2 had little effect on bacterial invasion or replication with either the WT or Δ*spvB* strains of STm (Fig. 7g and h). As detected for HeLa cells, STm infection of WT HEK293A cells induced significantly more LDH release in comparison to the Δ*spvB* strain and uninfected controls (Fig. 7i). However, LDH release was significantly reduced following STm infection of the HEK293A KO cells in comparison to HEK293A WT cells, with WT and Δ*spvB* STm-induced LDH release now no longer significantly different. This implies that SpvB-induced cell death is MAP4K/MST1/2-dependent (Fig. 7i). Morphologically, STm infection of HEK293A WT cells induced cell vacuolization (black arrows) and monolayer destruction/detachment (evident by visible plastic; Fig. S12), recapitulating the phenotypes observed following transient transfection. Consistent with LDH release, loss of MAP4K/MST1/2 or deletion of *spvB* rescued both cell vacuolization and monolayer destruction/detachment (Fig. S12), supporting a role for SpvB and MAP4Ks in STm-induced cell death. Finally, given the potential role of cathepsins in SpvB-induced cell death following transient expression, we tested their involvement in cell death induced by SpvB during STm infection. Treatment with ALLM and pepstatin A significantly inhibited LDH release from WT STm-infected cells. Of note, inhibition also had an additional effect on LDH release from Δ*spvB*-infected cells, suggesting that cathepsins mediate both SpvB-dependent and SpvB-independent cell death (Fig. 7j). Overall, SpvB-dependent cell death during STm infection followed a MAP4K/cathepsin-dependent pathway, mirroring cell death induced by ectopically expressed SpvB.

Although there was no significant difference in total bacterial CFUs between the WT and Δ*spvB* strains at 20 h post-invasion (Fig. 7h), we reproducibly detected an increase in the number of bacteria in the media of WT or Δ*spvB*+-infected cells in comparison to Δ*spvB*-infected cells. To quantify this, we repeated the infections, but this time enumerated the intracellular (whole-cell lysate) and extracellular (media only) bacteria separately. The majority of bacteria at 21 h post-invasion remained intracellular, with no significant difference in the number of intracellular bacteria between the different strains (Fig. 7k). However, deletion of *spvB* significantly reduced the number of bacteria recovered in the media (Fig. 7l). Therefore, a potential role of SpvB during infection could be to drive actin depolymerization and MAP4K/cathepsin-dependent cell death and lysis, promoting release of intracellular bacteria, which, in turn, may facilitate further infection of neighboring cells and bacterial dissemination within the gut.

## DISCUSSION

Many pathogens target the actin cytoskeleton through the secretion or translocation of actin depolymerizing toxins, ultimately triggering host cell death (18, 31, 80). In relation to *Salmonella* virulence, it was unclear whether SpvB-dependent cell death is a direct result of actin depolymerization. Through mutating actin at R177, the site of ADP-ribosylation, we obtained clear evidence that SpvB mART-dependent cell death is driven by the ribosylation of its only known host target: actin. Both morphologically and mechanistically, actin depolymerization-induced cell death was distinct from caspase-dependent apoptosis, with caspases found to be dispensable. This atypical mode of cell death instead proceeded via the Hippo signaling MAP4K family of stress kinases and JNK signaling and was partially rescued through cathepsin inhibition. Importantly, this atypical cell death occurred during infection, where endogenous levels of SpvB, translocated by STm, induced MAP4K-dependent cell death, facilitating the release of intracellular bacteria into the culture medium. Consistent with our findings, STm infection is reported to induce minimal mitochondrial membrane permeabilization (MOMP) with caspase-3 activity below the threshold required to induce apoptosis or DNA damage (81). Furthermore, Selkrig and colleagues recently reported cathepsin-dependent cell death during STm infection of macrophages, with cathepsins targeted to the nucleus (41). A role for SpvB in this process was not investigated, but it provides further evidence for the potential role of cathepsins in STm infection and cell death. Together, these experiments indicate a conserved mechanism of host cell death induced by actin depolymerizing agents. Therefore, our findings are likely to extend beyond STm and contribute to our understanding of the mechanisms of host cell death and the virulence of numerous important pathogens encoding mARTs or toxins that target actin. This includes *C. difficile,* which is reported to activate Hippo signaling (82) and is a leading cause of nosocomial infectious diarrhea and colitis (83, 84).

Morphologically, SpvB- and LatA-induced cell death was characterized by cell rounding, detachment, and vacuolization, with cells swelling and forming a singular large vacuole or a bubble-like morphology. Additionally, cells contained multiple nuclei, most likely due to G2/M cell cycle arrest, and were associated with markers of DNA damage, including condensation and fragmentation. These markers of nuclear damage are typical traits of apoptosis; however, there was a clear lack of cell shrinkage, blebbing, or apoptotic body formation: hallmarks of apoptosis. Alternatively, cell vacuolization during cell death is a morphological trait indicative of methuosis and paraptosis. Methuosis requires the actin cytoskeleton and was therefore ruled out as a potential pathway for actin depolymerization-induced cell death (67). Alternatively, paraptotic vacuoles are induced by mitochondrial and ER water-influx through Ca^2+^ release communicated through the Ca^2+^ uptake and release channels VDAC and IP_3_R (85). However, neither ER nor mitochondrial lumenal markers were associated with SpvB- or LatA-induced vacuoles. Despite attempts to characterize the vacuolar membrane, its origin remains unknown and did not stain for markers of the ER, lysosome, or mitochondria. Identifying the origin of these vacuoles remains difficult due to the fragile nature of the bubble-like morphology of these cells, which ruptured upon fixation, preventing immunofluorescent labeling and imaging, and limiting us to the expression of fluorescently tagged proteins or dyes. One limitation in our interpretation when comparing organelle staining between control cells and cells expressing SpvB was the considerable decrease in the cytoplasmic volume of SpvB-expressing cells. Increases in staining intensity or apparent aggregation of LAMP1-containing vesicles might be due to the increase in the concentration of vesicles in a smaller area. Despite these limitations, the morphological changes associated with actin depolymerization-induced cell death suggest an atypical cell death that is distinct from those previously described.

Our data implicate a MAP4K-JNK-dependent pathway that can be partially rescued through cathepsin inhibition. Cathepsin inhibition reduced both vacuolization and LDH release, implicating this family of proteins in SpvB-induced cell death; however, as LDH release was only partially rescued, our conclusions are limited at this stage. Partial rescue could be explained by the cytotoxic effect of pan-cathepsin inhibition, and that while SpvB-induced cell death was reduced, an alternative/additional pathway might have been triggered. Of note, an increase in basal LDH release for the GFP-transfected conditions was observed upon the addition of A/P (Fig. 6h). Alternatively, it could be that cathepsins play a partial but non-redundant role in SpvB-induced cell death, and an alternative parallel pathway simultaneously drives death through an unknown executioner(s). Although MAP4K, JNK, and cathepsins have been implicated in multiple modes of caspase-independent cell death, how they directly induce cell death remains unknown. JNK is implicated through either direct phosphorylation of key mediators of cell death (e.g., Bim/Bid) or through cJun-dependent transcriptional regulation of cell death inducers (75–78). Aligning with our data, one potential pathway is through JNK-induced lysosomal-dependent cell death (76, 86, 87), where in the case of massive cellular insult/trauma, lysosomal damage can result in overwhelming release of cathepsins and degradation of essential cellular proteins driving necrosis. A switch from caspase-dependent to caspase-independent cell death may in part be due to the amount and kinetics of an insult but could also be regulated by an accompanying rise in cytoplasmic pH during cell death, resulting in increased and broader cytoplasmic activity of the host cathepsins (88). Given the lack of caspase-dependent cell death, rescue through JNK inhibition and knockout, complete loss of Lysotracker staining, and partial rescue of cell death and inhibition of vacuolization with the addition of cathepsin inhibitors, our data favor a pathway where lysosome permeabilization and cathepsins contribute to cell death. However, it remains possible that lysosome permeabilization and cathepsin activity are concomitant events during cell death, rather than direct causes.

To address this and to fully delineate the complex pathway of actin depolymerization-induced cell death in general, remaining questions include the following: how do the large vacuoles form? how do the MAP4Ks sense actin depolymerization? what are the targets of JNK? is lysosomal permeabilization induced? and is this JNK-dependent? does the cytoplasmic pH change during SpvB-induced cell death? and finally, is there a concomitant increase in cytoplasmic cathepsin levels and if so, what are the targets leading to cell death?

SpvB mART activity is required for full virulence of STm, with mutation of the SpvB catalytic site or deletion of *spvB* rescuing intestinal barrier integrity, intestinal cell death, and limiting systemic infection following oral gavage in multiple animal models of infection (30, 53, 89). Consistent with these findings, we hypothesize that SpvB-induced cell death represents a process by which STm escapes the cell and inflammasome-dependent extrusion to facilitate disruption of the intestinal barrier integrity and dissemination. Future work should focus on the role of the key pathways identified in this study in animal models, including host cell Hippo signaling, MAP4Ks, JNK, and cathepsins. Unfortunately, Hippo signaling is essential for embryogenesis, ruling out the use of multiple MAPK knockouts. Furthermore, due to redundancy among this family, we were unable to identify a single MAP4K that controls cell death in response to actin depolymerization. Interestingly, Hippo signaling is not widely reported as a pathway targeted by bacterial virulence proteins, although there are a plethora of viral virulence factors and viruses that target and subvert this pathway, including herpes, Zika, and hepatitis C viruses (90). Unlike this study, viral infection is more commonly associated with inhibition of Hippo signaling, resulting in YAP/TAZ-dependent gene transcription (90). Given the critical role of MAP4Ks in SpvB-induced cell death, it is likely SpvB directly modulates downstream Hippo signaling independent of the induction of cell death and warrants further investigation.

More broadly, our findings can be extrapolated to non-infectious MAP4K-dependent cell death. Of note, Sertraline is reported to induce MAP4K4/JNK-dependent cell death of hepatic HepG2 cells. Intriguingly, the authors similarly found that while Sertraline induces caspase-3 activity, cell death is caspase-3 independent (72). As noted for actin depolymerizing agents, caspase activation has also been detected in MAP4K-dependent MN cell death and cardiomyocyte cell death (70, 71, 73). Again, whether cell death is mediated by caspases in these models remains to be investigated, and future studies investigating the potential role of cathepsins in MN, cardiomyocyte, and Sertraline-induced cell death would be justified.

In conclusion, our study identifies and maps out key components of a previously undescribed MAP4K/JNK-dependent cell death pathway driven by the direct disruption of the actin cytoskeleton by the mART SpvB and depolymerizing agent LatA, broadening our understanding of STm pathogenesis and virulence, and highlighting a novel role for host Hippo signaling MAP4Ks in bacterial infection and cell death.

## Data Availability

All data are included within the main text and Supplemental material.

## SUPPLEMENTAL MATERIAL

Supplemental material mbio.00655-26-s0001.pdf Fig. S1-S12 and Tables S1-S3.

ASM does not own the copyrights to Supplemental Material that may be linked to, or accessed through, an article. The authors have granted ASM a non-exclusive, world-wide license to publish the Supplemental Material files. Please contact the corresponding author directly for reuse.
