# Supplementary material for: MAP4Ks drive cell death in response to Salmonella SpvB-induced actin depolymerization
Source: mBio. 2026 May 20;17(6):e00655-26. doi: 10.1128/mbio.00655-26 (PMC13251412; doi:10.1128/mbio.00655-26)
Supplement: Supplemental material — Fig. S1-S12 and Tables S1-S3. [file mbio.00655-26-s0001.pdf]

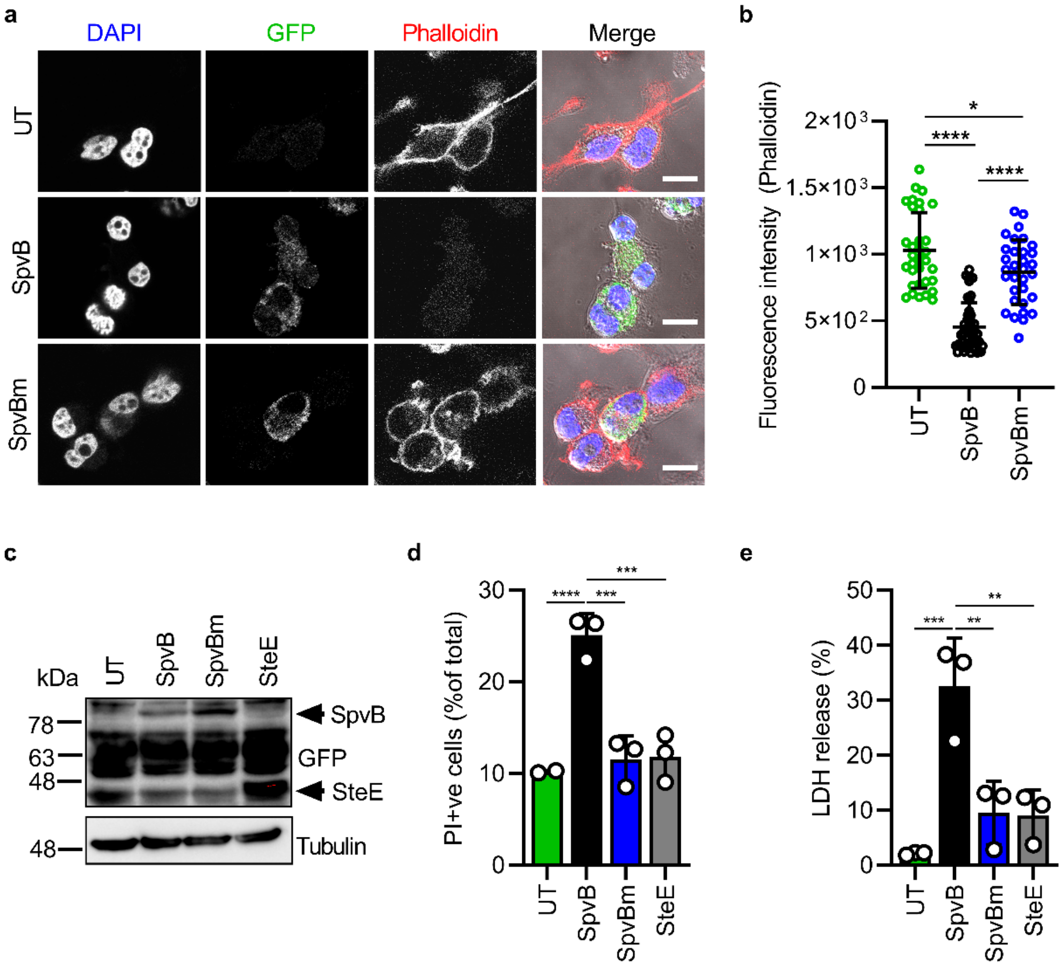

Supplementary Figure 1. SpvB-induced actin depolymerisation and cell death is mART-dependent. HeLa cells were transduced with lentiviral particles to express GFP-SpvB, GFP-SpvBm (catalytic dead), or GFP-SteE where stated, or left untransduced and analysed 24 h later. **(a)** Representative immunofluorescence staining of actin depolymerisation. Cells were stained with Phalloidin (polymerised actin; red) or DAPI (nuclei; blue) with GFP in green. Scale bar, 10 µm. **(b)** Mean fluorescence intensity of polymerised actin (Phalloidin). **(c)** In parallel, cell lysates were analysed by SDS-PAGE and immunoblotting with anti-GFP and anti-tubulin antibodies to determine relative protein expression. Arrows show GFP-SpvB and GFP-SteE. **(d)** Cell death was measured by Flow cytometry (% PI positive) and **(e)** LDH release**.** Representative of 3 independent experiments. Statistical significance was analysed by One-way ANOVA with Tukey's multiple comparison post-test. p<0.05 = *, p<0.01 = **, p<0.001 = *** and p<0.0001 = ****.

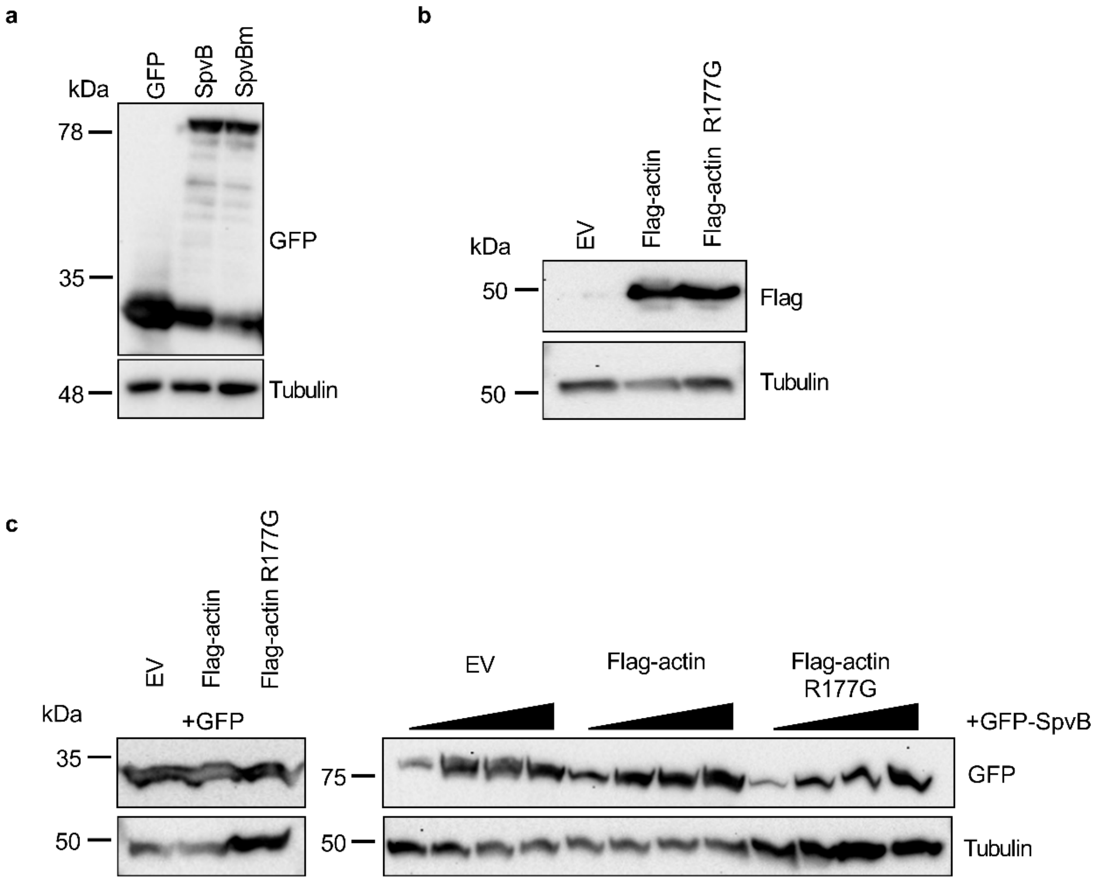

Supplementary Figure 2. Relative expression of GFP-tagged constructs. (a) HeLa cells were transfected with pEGFP-N1, ptCMV-GFP-SpvB or ptCMV-GFP-SpvBm. Twenty-four hours post-transfection cell lysates were analysed by SDS-PAGE and immunoblotting with anti-GFP and anti-tubulin antibodies to determine relative protein expression. Representative of three independent experiments. **(b)** Representative immunoblot of Flag-tagged protein expression relative to tubulin following HEK293A transfection with pCDNA4/TO to express Flag-actin or Flag-actin R177G or transfection with empty vector (EV) as a control and Zeocin selection. **(c)** HEK293-EV (EV), HEK293-Flag-actin and HEK293-Flag-actin R177G cell lines were transfected with pEGFP-N1 or an increasing dose of ptCMV-GFP-SpvB (25, 50, 75 or 150 ng) and cell lysates were analysed by SDS-PAGE and immunoblotting of GFP and tubulin. Representative of three independent experiments.

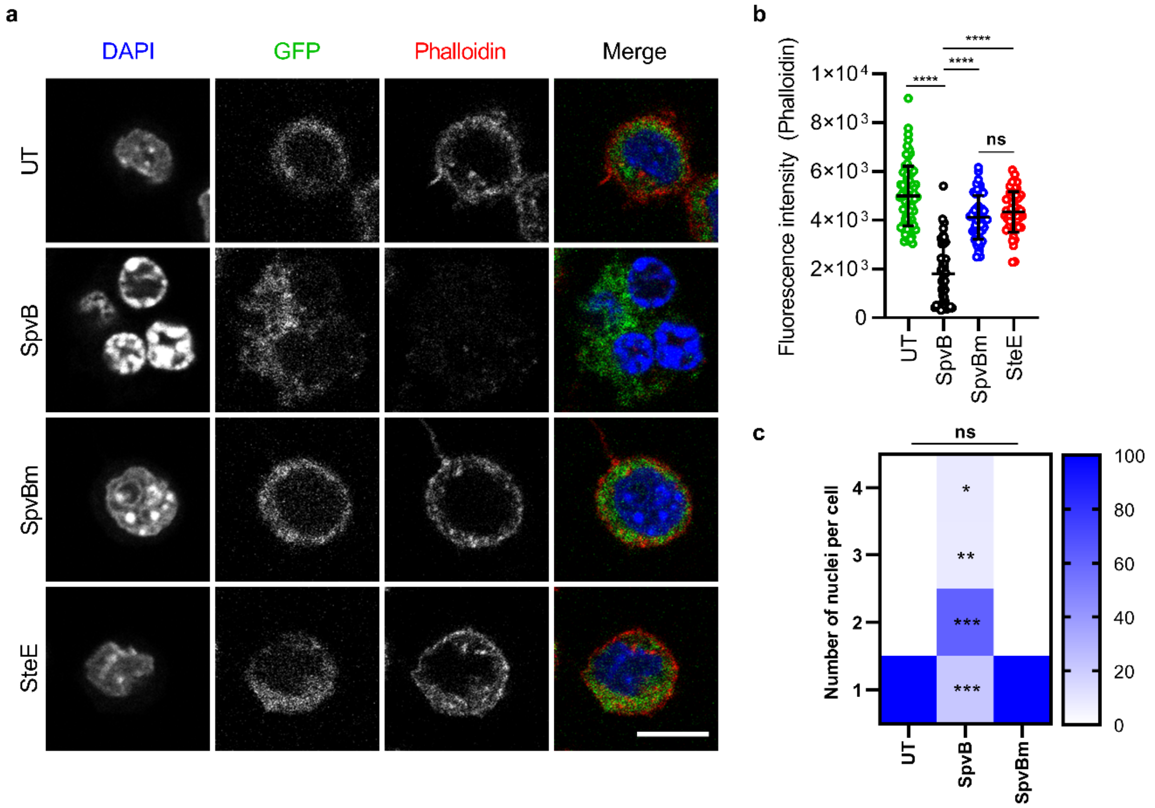

Supplementary Figure 3. SpvB induces actin depolymerisation and nuclei accumulation and damage in macrophages (a-c) J774A.1 mouse macrophages were transduced with lentiviral particles to express GFP-SpvB, GFP-SpvBm (catalytic dead), GFP-SteE or left untransduced and analysed 48 h later. **(a)** Representative immunofluorescence staining of actin depolymerisation. Cells were stained with Phalloidin (polymerised actin; red) or DAPI (nuclei; blue) with GFP in green. Scale bar, 10 µm. **(b)** Mean fluorescence intensity of polymerised actin (red). Statistical significance was analysed by One-way ANOVA with Tukey's multiple comparison post-test. **(c)** Number of nuclei per cell. Representative of two independent experiments. Statistical significance was analysed by Two-way ANOVA with Tukey's multiple comparison post-test. Significance shown for SpvB vs UT and SpvBm. $p<0.05$ = *, $p<0.01$ = **, $p<0.001$ = *** and $p<0.0001$ = ****.

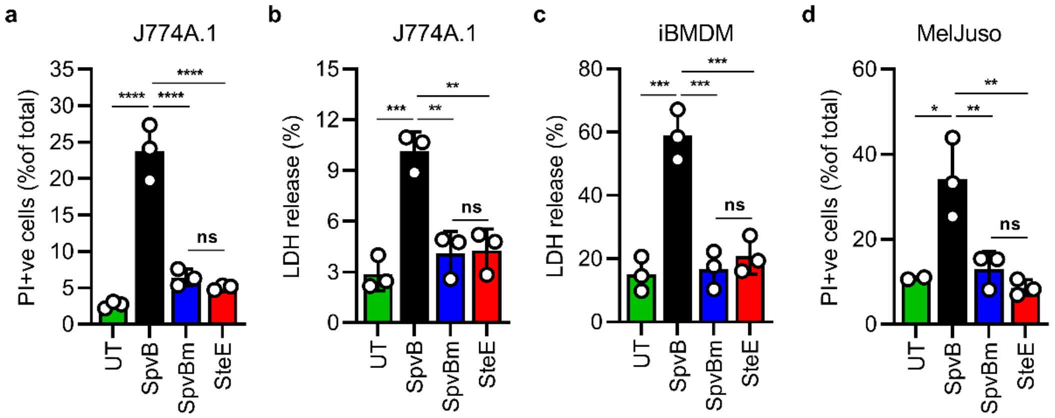

Supplementary Figure 4. SpvB induces cell death in multiple cell types. (a-b) J774A.1 mouse macrophages were transduced with lentiviral particles to express GFP-SpvB, GFP-SpvBm (catalytic dead), GFP-SteE or left untransduced and analysed 48 h later. **(a)** Cell death was measured by Flow cytometry (% PI positive). Representative of two independent experiments. **(b)** Cell death was measured by LDH release assay. Representative of three independent experiments. **(c)** Mouse iBMDM were transduced with lentiviral particles to express GFP-SpvB, GFP-SpvBm (catalytic dead), GFP-SteE or left untransduced. At 48 h post-transduction cell death was measured by an LDH release assay. Representative of two independent experiments. **(d)** MelJuso cells were transduced with lentiviral particles to express GFP-SpvB, GFP-SpvBm (catalytic dead), GFP-SteE or left untransduced. At 72 h post-transduction cell death was analysed by flow cytometry (% PI positive). Representative of three independent experiments. Statistical significance was analysed by One-way ANOVA with Tukey's multiple comparison post-test. p<0.05 = *, p<0.01 = **, p<0.001 = *** and p<0.0001 = ****.

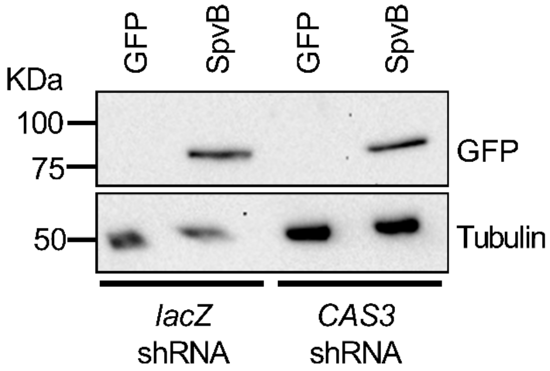

Supplementary Figure 5. Knockdown of *Cas3* does not affect expression of GFP-tagged constructs. Relative levels of SpvB expression in the HeLa Cas3-shRNA and HeLa LacZ-shRNA cell lines was determined by SDS-PAGE and immunoblotting with antibodies against GFP and tubulin. Representative of three independent experiments.

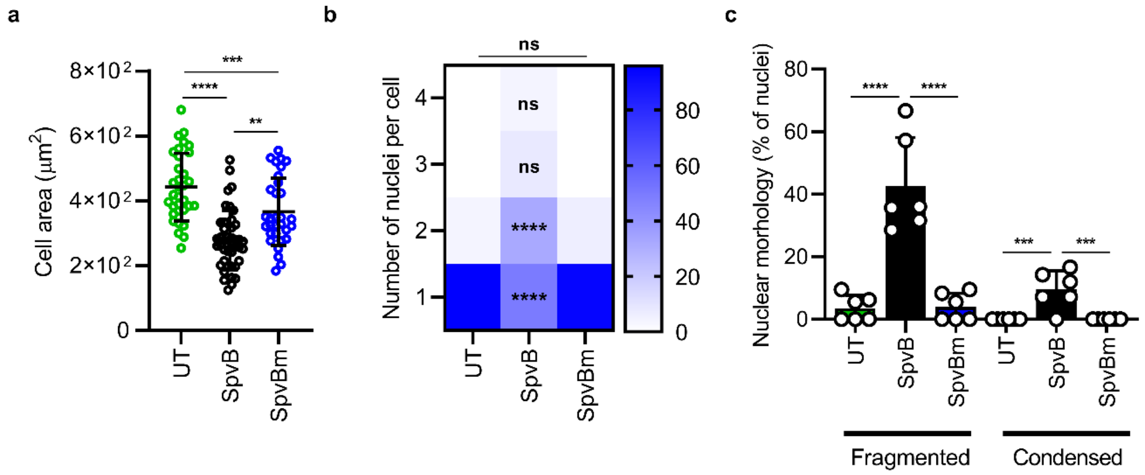

Supplementary Figure 6. Cell morphological changes are dependent on the mART activity of SpvB. (a-c) HeLa cells were transduced with lentiviral particles to express GFP-SpvB or GFP-SpvBm (catalytic dead) or left untransduced and analysed 24 h later. Cells were stained as in Figure 1a and changes in cell size and nuclei number and morphology were enumerated. **(a)** Average cell area. Statistical significance was analysed by One-way ANOVA with Tukey's multiple comparison post-test. **(b)** Number of nuclei per cell. Statistical significance was analysed by Two-way ANOVA with Tukey's multiple comparison post-test. Significance shown for SpvB vs GFP and SpvBm. **(c)** Percentage of fragmented or condensed nuclei. Representative of three independent repeats. Statistical significance was analysed by One-way ANOVA with Tukey's multiple comparison post-test. $p<0.05$ = *, $p<0.01$ = **, $p<0.001$ = *** and $p<0.0001$ = ****.

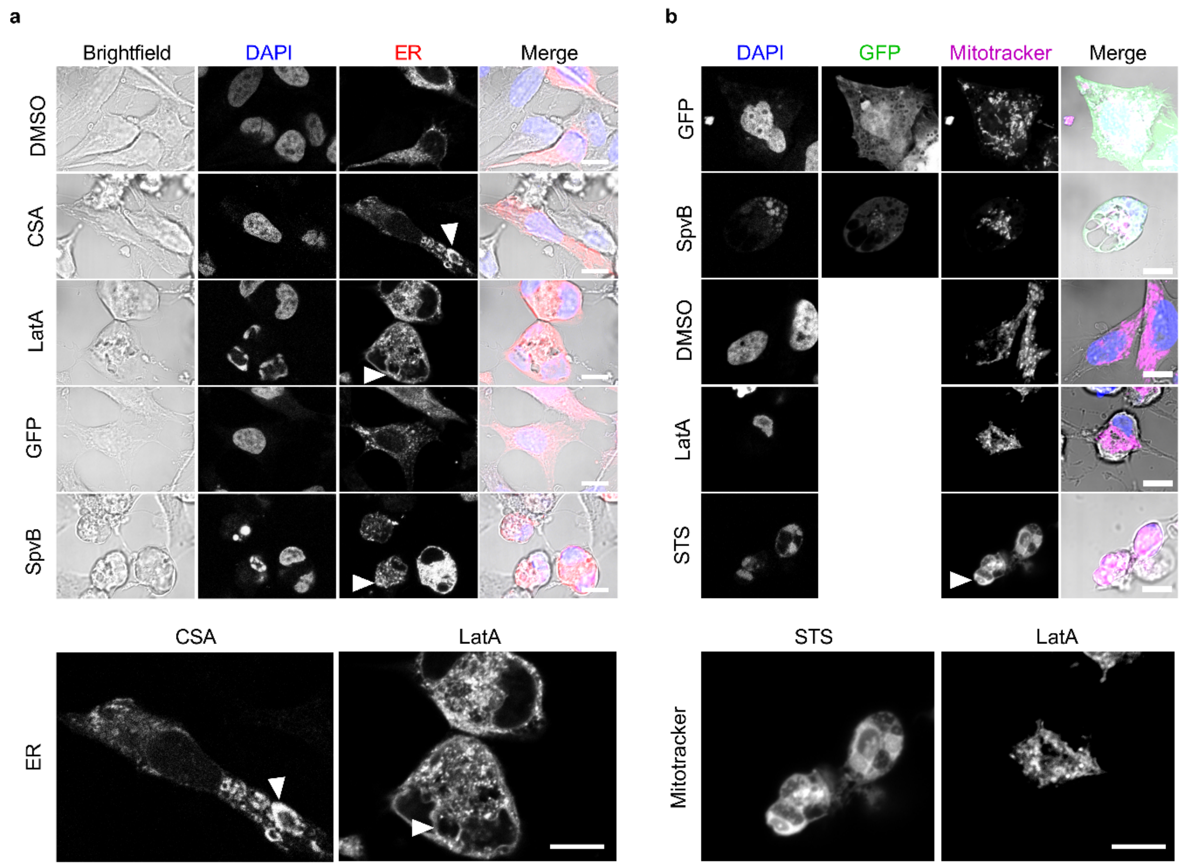

Supplementary Figure 7. SpvB and latrunculin A induced vacuoles are not mitochondrial or ER derived. HeLa cells were co-transfected with the ER lumen marker (KDEL mCherry) with pEGFP-N1 or ptCMV-GFP-SpvB or transfected with ER lumen marker and treated with DMSO, 9.6 µM Latrunculin A (LatA) or 40 µM Cyclosporin A (CSA) and analysed 24 h post-transection and 18 h post-treatment, respectively. **(a)** Representative confocal immunofluorescence images of fixed attached cells with ER (mCherry), GFP (green) and nuclei stained with DAPI (blue). White arrows indicate vacuoles. Lower panels show 2 x further magnification and co-localisation of ER marker with vacuoles. Scale bar, 10 µM. White arrows indicate vacuoles. Representative of three independent repeats. **(b)** Representative immunofluorescence images of fixed attached cells with Mitotracker (red), DAPI (blue) and with GFP (green) 24 h post-transfection of HeLa cells with pEGFP-N1 or ptCMV-GFP-SpvB or 18 h post-treatment with DMSO, 9.6 µM LatA or 1 µM Staurosporine (STS). One hour prior to fixation cells were stained with Mitotracker and DAPI. White arrows indicate cytoplasmic staining with Mitotracker. Scale bar, 10 µM. Lower panels show 2 x further magnification to show diffuse Mitotracker staining upon STS treatment in comparison to LatA treated cells. Scale bar, 10 µM. Representative of three independent experiments.

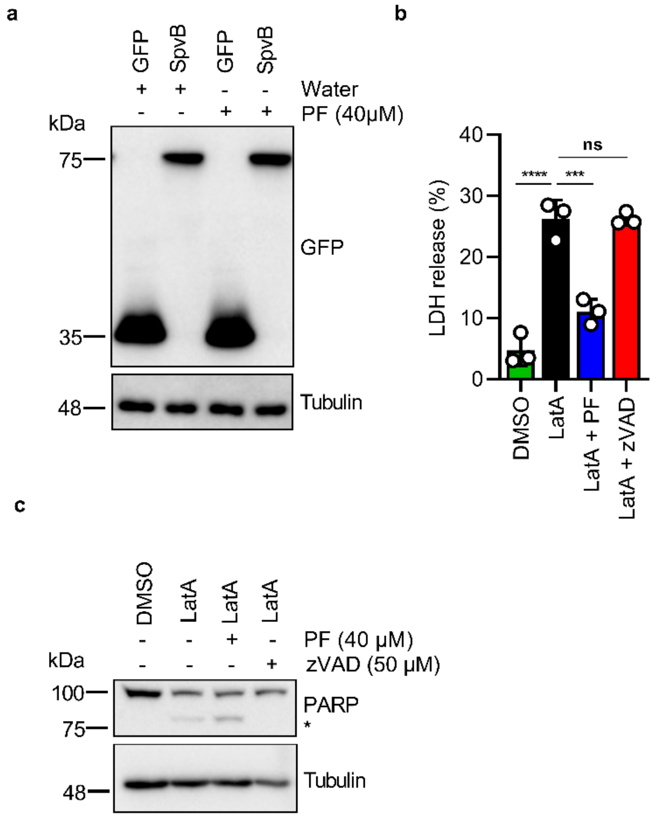

Supplementary Figure 8. MAP4K4/6/7 inhibition rescues SpvB and Latrunculin A dependent cell death but not PARP-1 cleavage. HeLa cells were transfected with pEGFP-N1 or ptCMV-GFP-SpvB with or without the addition of 40 µM PF-06260933 (PF) at 6 h post-transfection. Cell lysates were analysed 24 h post-transfection by SDS-PAGE and immunoblotting for GFP and tubulin. **(a)** Representative immunoblot showing relative GFP and GFP-SpvB expression. Representative of three independent experiments. **(b-c)** HeLa cells were treated with DMSO or 9.6 µM Latrunculin A (LatA) with or without DMSO vehicle, 40 µM PF or 50 µM z-VAD-FMK. **(b)** LDH release. Representative of three independent experiments. Statistical significance was analysed by Two-Way ANOVA with Tukey's multiple comparison post-test. **(c)** In parallel, cell lysates were analysed by SDS-PAGE and immunoblotting with anti-PARP-1 and anti-tubulin antibodies. Representative of three independent experiments. *, cleaved PARP-1; ns, non-significant. $p<0.05$ = *, $p<0.01$ = **, $p<0.001$ = *** and $p<0.0001$ = ****.

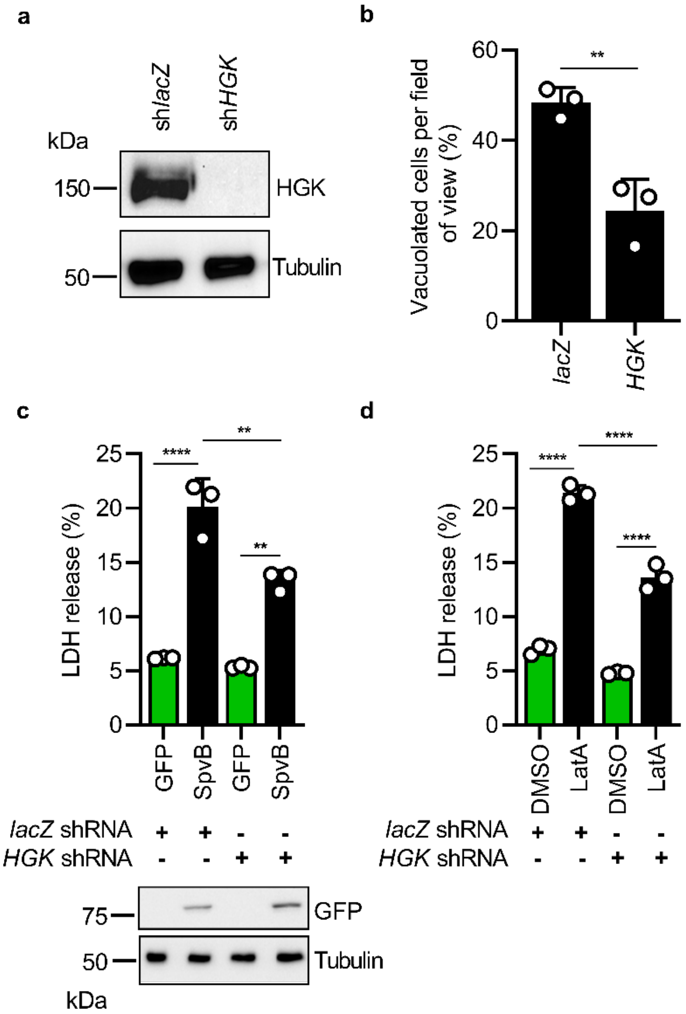

Supplementary Figure 9. Knockdown of MAP4K4 partially rescues actin depolymerisation induced cell death. HeLa cells were transduced to express YFP-tagged miR30E shRNA targeting *HGK (MAP4K4)* or a non-targeting (*lacZ*) control. **(a)** Knockdown efficiency was determined by SDS-PAGE and immunoblotting of cell lysates with antibodies for HGK and tubulin. **(b-d)** HeLa HGK-shRNA and HeLa LacZ-shRNA cell lines were transfected with pEGFP-N1 or ptCMV-GFP-SpvB or treated with 9.6 μM Latrunculin (LatA) or DMSO control where stated. **(b)** Enumeration of detached vacuolised cells. Representative of three independent repeats. Statistical significance analysed by unpaired Student's T-test. **(c-d)** Cell death was analysed by an LDH release assay at 18 h post-treatment and 24 h post-transfection. Average of three independent repeats. In parallel, relative expression of SpvB in HeLa HGK-shRNA and HeLa LacZ-shRNA cell lines was analysed by SDS-PAGE and immunoblotting with anti-GFP and anti-tubulin antibodies. Representative of three independent experiments. Statistical significance was analysed by Two-Way ANOVA with Tukey's multiple comparison post-test. $p<0.05$ = *, $p<0.01$ = **, $p<0.001$ = *** and $p<0.0001$ = ****.

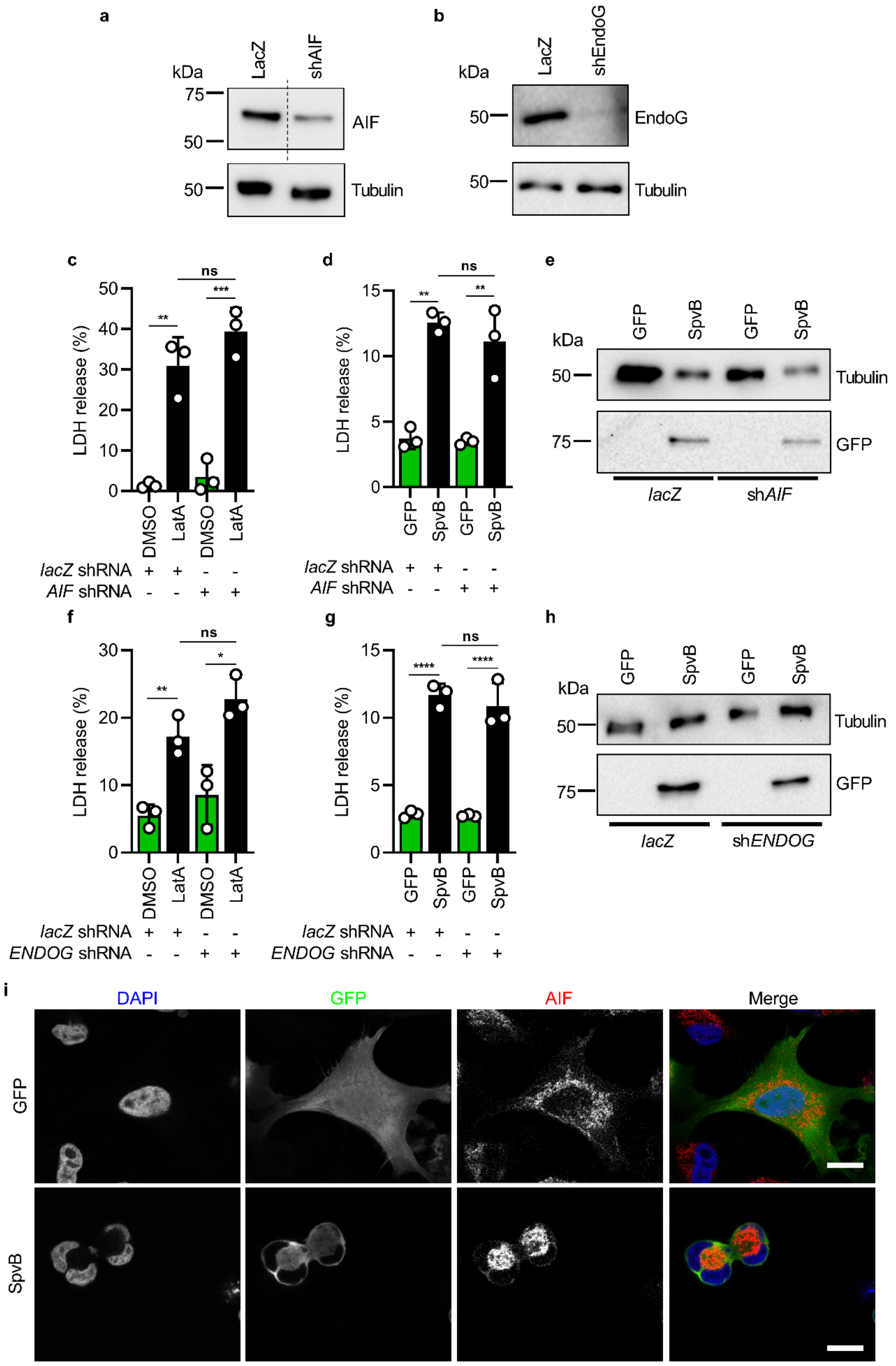

Supplementary Figure 10. Cell death is EndoG and AIF-independent. HeLa cells were transduced to express YFP-tagged miR30E shRNA targeting *ENDOG*, *AIFM1* or a non-

targeting (*lacZ*) control. **(a-b)** Knockdown efficiency was determined by SDS-PAGE and immunoblotting of cell lysates with antibodies for EndoG, AIF and tubulin. Dotted line indicates cropped image. **(c-e)** HeLa AIF-shRNA and HeLa LacZ-shRNA cell lines were transfected with pEGFP-N1 or ptCMV-GFP-SpvB or treated with 9.6 µM Latrunculin A (LatA) or DMSO control where stated. **(c-d)** Cell death was analysed by an LDH release assay at 18 h post-treatment and 24 h post-transfection. Average of three independent repeats. Statistical significance analysed by Two-Way ANOVA with Tukey's multiple comparison post-test. **(e)** In parallel, relative expression of SpvB in HeLa AIF-shRNA and HeLa LacZ-shRNA cell lines was analysed by SDS-PAGE and immunoblotting with anti-GFP and anti-tubulin antibodies. Representative of three independent experiments. **(f-h)** HeLa EndoG-shRNA and HeLa LacZ-shRNA cell lines were transfected with pEGFP-N1 or ptCMV-GFP-SpvB or treated with 9.6 µM Latrunculin or DMSO control where stated. **(f and g)** Cell death was analysed by an LDH release assay at 18 h post-treatment and 24 h post-transfection. Average of three independent repeats. Statistical significance analysed by Two-Way ANOVA with Tukey's multiple comparison post-test. **(h)** In parallel, relative expression of SpvB in HeLa EndoG-shRNA and HeLa LacZ-shRNA cell lines was analysed by SDS-PAGE and immunoblotting with anti-GFP and anti-tubulin antibodies. Representative of three independent experiments. **(i)** Representative confocal immunofluorescence images of AIF staining in HeLa cells 24 h post-transfection with pEGFPN1 or ptCMV-GFP-SpvB. Cells were stained for AIF (red) and GFP (green) with nuclei stained with DAPI (blue). Representative of three independent experiments. Scale bar, 10 µm. $p<0.05$ = *, $p<0.01$ = **, $p<0.001$ = *** and $p<0.0001$ = ****.

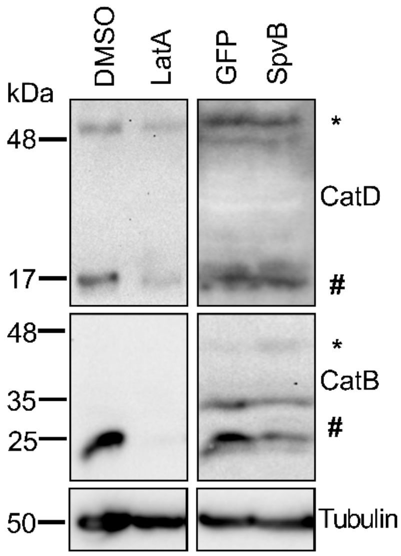

Supplementary Figure 11. Latrunculin A treatment or SpvB expression reduces cathepsin processing. HeLa cells were transfected with pEGFP-N1 or ptCMV-GFP-SpvB or treated with 9.6 µM Latrunculin A (LatA) or DMSO control, where stated, and analysed 24 h post-transfection and 18 h post-treatment, respectively. Cell lysates were analysed by immunoblotting using anti-cathepsin B, anti-cathepsin D and anti-tubulin antibodies. *, cathepsin proform; #, active cathepsin. Two bands predicted due to glycosylated or non-glycosylated cathepsin B. Representative of three independent experiments.

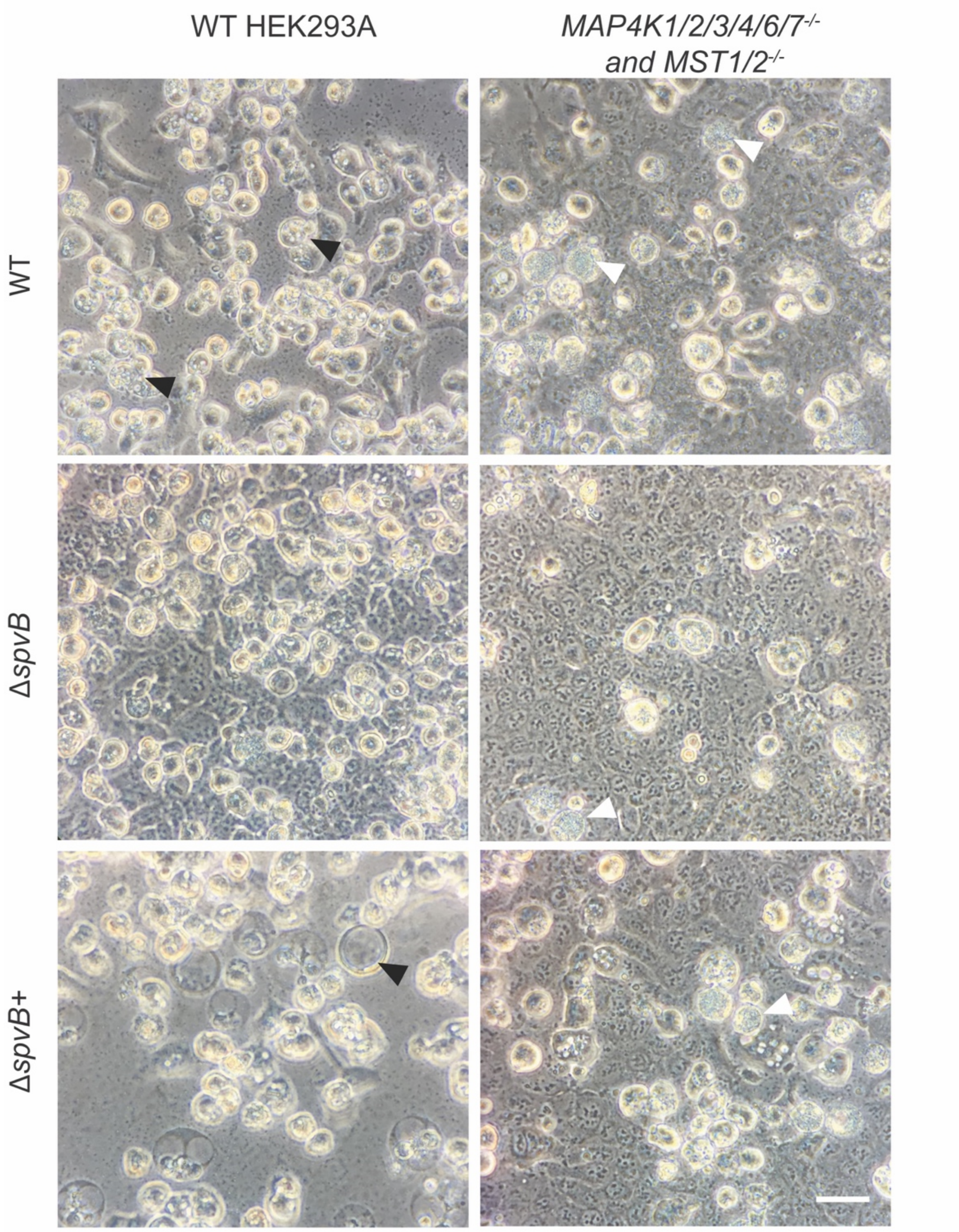

Supplementary Figure 12. SpvB-dependent vacuolisation and monolayer destruction is MAP4K-dependent. HEK293A or HEK293A KO (*MAP4K1/2/3/4/6/7*$^{-/-}$ and *MST1/2*$^{-/-}$) cells were infected with *Salmonella* SL1334 strains WT pACYC-EV, Δ*spvB* pACYC-EV or Δ*spvB* pACYC-*spvB* at an MOI of 50. Representative light micrographs were taken at 20 h post-infection. Scale bar, 25 µm. Representative of three independent experiments. Black arrows, vacuolisation; white arrows, rounded cells highly infected with *Salmonella*.

.

**Parental plasmid**	**Description**		**Reference/Source**
**pcDNA4/TO-**	Mammalian expression vector under the control of a CMV promoter, Amp^r, Zeo^r		Invitrogen
	**Primer**	**RE**	
pcDNA4/TO-Flag-actin	GCGGATCCATGGACTACAAAGACGATGACGACAAGATGGATGATGATATCGCCGC<br>GCGAATTCCTAGAAGCATTTGCGGTGGA	BamHI<br>EcoRI	This study
pcDNA4/TO-Flag Actin R177A	GCGGATCCATGGACTACAAAGACGATGACGACAAGATGGATGATGATATCGCCGC<br>GCGAATTCCTAGAAGCATTTGCGGTGGA	BamHI<br>EcoRI	This study
**ptCMV-GFP**	pEGFPN1 derivative mammalian expression vector with an N-terminal GFP tag under the CMV promoter, Kan^r		Teresa Thurston, Imperial College London, (1)
	**Primer/Template**	**RE**	
ptCMV-GFP-SpvB	N/A		Gift, Teresa Thurston
ptCMV-GFP-SpvBm	pLTREK-GFP-SpvBm	EcoRI<br>XhoI	This study
**pLTREK-GFP**	Lentiviral expression plasmid with an N-terminal GFP tag under the Ter-ON promoter, Amp^r and $Puro^r$		Avinash Shenoy, Imperial College London (2)
	**Primer**	**RE**	
pLTREK-GFP-SpvB	AACCGTCAGATCGCCTGATATCGCCACCATGGTGAGCAAGGGCGAGG<br>TGTACAGCTCGTCCATGCTAGCTCATGAGTTGAGTACCCTCATGTTTA	EcoRI<br>XhoI	This study
pLTREK-GFP-SpvBm	AACCGTCAGATCGCCTGATATCGCCACCATGGTGAGCAAGGGCGAGG<br>TGTACAGCTCGTCCATGCTAGCTCATGAGTTGAGTACCCTCATGTTTA	EcoRI<br>XhoI	This study
pLTREK-GFP-SteE	AACCGTCAGATCGCCTGATATCGCCACCATGGTGAGCAAGGGCGAGG<br>GGAACATGCTGATCATTTAAGAC	EcoRI	This study
**pCMV-PACK**	Packaging plasmid for lentivirus production with HIV Gag, Pol, Rev and Tat under the CMV promotor, Amp^r		Gift, Dr. H. Laman
**pCMV-ENV**	VSV-G pseudotyped envelope protein under the CMV promoter for lentivirus production, Amp^r		Gift, Dr. H. Laman
**pMXs-IP-YFP**	Retroviral expression plasmid with YFP under the CMV promoter, Amp^r and $Puro^r$		Invitrogen
	**Primer**	**RE**	
pMXs-IP-YFP miR30E-*CASP3*	tcgagaaggtatattgctgttgacagtgagcgCATCCCTGGACAACAGTTATAAtagtgaagccacagat<br>cttgaagtccgaggcagtaggcaTATCCCTGGACAACAGTTATAAtacatctgtggcttcactaTTATAA	EcoRI<br>XhoI	This study
pMXs-IP-YFP miR30E-*AIF*	tcgagaaggtatattgctgttgacagtgagcgCCGTTCAGCTACCCTGACTTTCtagtgaagccacagat<br>cttgaagtccgaggcagtaggcaACGTTCAGCTACCCTGACTTTCtacatctgtggcttcactaGAAAGT	EcoRI<br>XhoI	This study
pMXs-IP-YFP miR30E-*HGK*	tcgagaaggtatattgctgttgacagtgagcgACCTACATCAGTAGCATATATTtagtgaagccacagat<br>cttgaagtccgaggcagtaggcaGCCTACATCAGTAGCATATATTtacatctgtggcttcactaAATATA	EcoRI<br>XhoI	This study
pMXs-IP-YFP miR30E-*EndoG*	tcgagaaggtatattgctgttgacagtgagcgACCTGGAACAACCTGGAGAAATtagtgaagccacagat<br>cttgaagtccgaggcagtaggcaGCCTGGAACAACCTGGAGAAATtacatctgtggcttcactaATTTCT	EcoRI<br>XhoI	This study
**pX330**	Plasmid for expression of human-codon-optimized *Sp*Cas9 protein and a guide RNA		Addgene, 42230
	**Primer**	**RE**	
pX330-MAPK8	Pair 1 CACCgTCGCTACTACAGAGCACCCG; AAACCGGGTGCTCTGTAGTAGCGAc<br>Pair 2 CACCgAACACCCGTACATCAATGTC; AAACGACATTGATGTACGGGTGTTc	BbsI	This study
pX330-MAPK9	Pair 1 CACCGAAGTTAGTGCACGCTGTCC; AAACGGACAGCGTGCACTAACTTC<br>Pair 2 CACCGTACCGTGTCACCACGTAAG; AAACCTTACGTGGTGACACGGTAC	BbsI	This study
pX330-MAPK10	Pair 1 CACCgTTACCGCCTCCACTTCGGCT; AAACAGCCGAAGTGGAGGCGGTAAc<br>Pair 2 CACCgAAGGTGAGTCCCGCATACTT; AAACAAGTATGCGGGACTCACCTTc	BbsI	This study

Table S1. Plasmids used in this study. Amp^r, Ampicillin resistance; $Puro^r$, Puromycin resistance; Zeo^r, Zeocin resistance.

**Primer**	**Sequence 5'3**
**1**	aaccgtcagatcgcctgatatcgccaccatggtgagcaagggcgagg
**2**	gagggaaaagcatgtctgcctctccc
**3**	gggagaggcagacatgcttttccctc
**4**	tgtacagctcgtccatgctagctcatgagttgagtaccctcatgttta
**5**	gcacattttaagggagacgcagacatgcttttccctccaaatac
**6**	gtatttggagggaaaagcatgtctgcgtctcccttaaaatgtgc
**7**	tgtacaagtccggactcagatgatcgacttcttaacccaacagaaggctcgagaaggtatattgctg
**8**	gcggccgcggcgcgccggccgaattctagccccttgaagtccgaggcagtagg
**9**	gcggatccatggactacaaagacgatgacgacaagatggatgatgatatcgccgc
**10**	ccagccaggtccagacccaggatggcatggg
**11**	cccatgccatcctgggtctggacctggctgg
**12**	gcgaattcctagaagcatttgcggtgga
**13**	atgttgatactaaatggtttttcatctgccactttagcggtgtaggctggagctgcttc
**14**	ctatgagttgagtaccctcatgtttattattctttttatccatatgaatatcctccttagt
**15**	cgttccgagcagaaagttaaag
**16**	gagcgacgtcattgttcagg

Table S2: Primers used in this study

Strain Name	Parental/description	Resistance	Reference/Source
WT SL1334 pACYC-184-EV	Str^R*hisG rpsL xyl*	Cm^r	(3)
Δ*spvB* SL1344 pACYC184-EV	SL1344	Kan^r, Cm^r	This study
Δ*spvB* SL1344 pACYC184-*spvB*	SL1344	Kan^r, Cm^r	This study

Table S3. Strains used in this study. EV, empty vector; Kan^r, Kanamycin resistance; Cm^r, Chloramphenicol resistance.

Supplementary references

1. Günster RA, Matthews SA, Holden DW, Thurston TLM. 2017. SseK1 and SseK3 Type III Secretion System Effectors Inhibit NF-κB Signaling and Necroptotic Cell Death in Salmonella-Infected Macrophages. Infect Immun 85.

2. Eldridge MJG, Sanchez-Garrido J, Hoben GF, Goddard PJ, Shenoy AR. 2017. The Atypical Ubiquitin E2 Conjugase UBE2L3 Is an Indirect Caspase-1 Target and Controls IL-1β Secretion by Inflammasomes. Cell Rep 18:1285–1297.

3. Hoiseth SK, Stocker BA. 1981. Aromatic-dependent Salmonella typhimurium are non-virulent and effective as live vaccines. Nature 291:238–239.
